# A Model for Rapid Innovation for Engagement, Enrollment, and Data and Sample Collection in a Diverse Cohort Study: Insights from *All of Us* Participant Labs

**DOI:** 10.1016/j.mcpdig.2025.100227

**Published:** 2025-05-21

**Authors:** Janna Ter Meer, Jessica Chen, Romina Foster-Bonds, Andrea Goosen, Gayle Valensky, Ethan Dinh-Luong, Rachele Peterson, Geoffrey Ginsburg, Chris Lunt, Vik Kheterpal, Eric Topol, Allison Mandich, Yentram Huyen, Julia Moore Vogel

**Affiliations:** a Scripps Research Translational Institute, Scripps Research, La Jolla, CA; b All of Us Research Program, Division of Program Coordination, Planning, and Strategic Initiatives, Office of the Director, National Institutes of Health, Bethesda, MD; c CareEvolution, Ann Arbor, MI

## Abstract

**Objective:**

To improve engagement and retention of a cohort that reflects the US population within the *All of Us* Research Program, we created and implemented an innovation infrastructure and initiatives.

**Participants and Methods:**

*All of Us* participant laboratories (APLs) established innovation-specific processes to rapidly ideate, select, implement, and evaluate cost-effective innovative initiatives, while mitigating risks. This was done within 4 priority areas: accelerating enrollment, enhancing engagement and retention, improving biospecimen collection, and broadening data types. Participants within the *All of Us* Research Program were engaged in this research between April 6, 2022 and May 6, 2024.

**Results:**

We present a summary of APL processes and portfolio along with 5 specific initiatives that rapidly tested innovative ways to increase task completion and broaden biospecimen submission accessibility. Each initiative’s cost–benefit profile was evaluated by a committee of program leadership. Findings include the following: (1) offering compensation increased task completion, the degree of which was dependent on the context and amount of compensation; and (2) adding evening and weekend blood donation appointment times and distributing saliva collection kits through community partners increased donations from participants who have been historically underrepresented in biomedical research. On average, program staff predicted initiative effect sizes would be more than double their actual effect.

**Conclusion:**

We found that large research studies can rapidly innovate to meet program goals, including a focus on diversity. We identified specific strategies and tactics to improve health research engagement and retention, with a focus on historically underrepresented in biomedical research communities, which can be used by numerous health research studies.

Health research studies struggle to recruit and retain participants who reflect the populations they aim to serve.^1^, ^2^, ^3^ Further, traditional models of research move slowly. A growing number of research and clinical studies give participants the option to interact remotely.^4^ The *All of Us* Research Program (*All of Us*) is one of the first to attempt sustaining engagement of a diverse population for over 10 years through primarily remote participation.^5^

*All of Us* created a health research database,^5^ with 45% participants who are historically underrepresented in biomedical research based on race and/or ethnicity (UBR R/E) and over 80% UBR overall.^6^ UBR overall means historically underrepresented in biomedical research.^7^

Crucially, the program enables eligible individuals across the United States to participate. As of April 2025, over 860,000 participants have consented to share data in a research platform fueling over 17,000 studies.^8^

The program is working toward recruiting at least 1 million participants who reflect the diversity of the United States, and 6 years after launching, historically underrepresented in biomedical research (UBR) retention rates are lower than retention rates for participants who have been historically represented in biomedical research (RBR). For example, 34% (n=195,854) of UBR R/E participants completed a program activity in the last 18 months, compared with 54% (n=312,447) of participants who are historically represented in biomedical research based on their race and/or ethnicity. To fulfill the program’s goals of accelerating medical breakthroughs and reducing health disparities, improvement is needed.

We created *All of Us* participant laboratories (APL) to introduce rapid-cycle innovation. We ideated, selected, implemented, and evaluated cost-effective innovation initiatives, whereas reducing risk of large-scale delays or costly failures by starting with small-scale efforts. Each initiative aligned with program priorities (Supplemental Figure 1) and had clear success criteria. This enabled evaluation of performance and abandonment of initiatives with null or negative effects. Below are innovation processes and efforts to scale initiatives, along with 5 initiatives and results that can have a substantial impact toward meeting research program goals, focusing on diversity.

## Participants and Methods

*All of Us* participant laboratories had 4 priorities: (1) accelerating enrollment, (2) enhancing engagement and retention, (3) improving biospecimen collection, and (4) broadening data types (including digital health technologies, surveys, and electronic health records [EHRs]). Annual targets and quarterly reviews helped balance the portfolio across program goals, project size, and risk (Supplemental Figure 2 and Supplemental Table 3).

*All of Us* participant laboratories were a collaboration between National Institutes of Health (NIH) staff and Scripps Research. Scripps explored concepts and assessed feasibility, then implemented and evaluated each initiative. A program leadership committee, comprised the leads of the program’s health equity, innovation, scientific, and technology divisions within NIH and 1 of Scripps’s coprincipal investigators, was responsible for determining which proposed initiatives would use APL resources and whether initiatives should scale to the broader consortium. This committee devised entry and exit criteria that all initiatives were evaluated against (Supplemental Tables 1 and 2, available online at https://www.mcpdigitalhealth.org/). The committee reviewed materials and provided decisions within 1 week and advocated for innovation within the consortium.

Initiatives started with developing a problem statement, based on input from consortium partners; reviewing literature; and running landscape analyses. Next, we considered multiple potential solutions and their tradeoffs. We often used user testing and consulted with Scripps’s Virtual Advisory Team, a group of 21 *All of Us* participants (77% UBR R/E, n=16) to solicit feedback. Third, we designed initiative-specific evaluations, often randomized controlled trials (RCTs) to maximize internal validity,^9^ although we used feasibility testing, quasi-experimental, or retrospective analyses to address ethical, logistical, and/or financial constraints.^10^ Across 12 interventions tested via RCT, we collected 173 predictions from program staff about expected effects to benchmark findings and increase accountability and learning.^11^ All human subjects research in this manuscript was approved by the *All of Us* institutional review board (IRB). Finally, we established processes to support a culture of innovation, where every idea would be thoughtfully considered.

Participants were engaged in the following initiatives during the following timelines (approved by the *All of Us* IRB under the following project names): (1) streamlining participant-mediated EHR sharing: April 6, 2022 to January 10, 2023; (2) financial compensation for donating a biospecimen: April 24, 2023 to January 18, 2024; (3) symbolic rewards and financial compensation for general engagement: December 14, 2023 to May 6, 2024; (4) expanding biospecimen collection appointment times: May 19 to July 14, 2022; and (5) distributing saliva kits via community partners: June 1 to September 15, 2023.

## Results

### Innovation Processes and Portfolio

*All of Us* participant laboratories–specific processes were developed to support rapid review and implementation. The program leadership committee met the goal of providing decisions within 1 week of receiving materials. Initiative-specific timelines were often lengthened by delayed IRB reviews (including delays in establishing an APL umbrella protocol under which subsequent APL studies could be housed and APL initiatives being deprioritized relative to the program’s other goals). Establishing the APL umbrella protocol, with each initiative as an appendix, succeeded at reducing IRB review timelines when the IRB had bandwidth to complete reviews. Meetings with the IRB to discuss innovation goals and IRB concerns before submitting the novel initiatives helped reduce stipulations. Another source of delays was recruitment for APL studies; owing to technical constraints, recruitment from the entire consortium was not always possible, and some initiatives included only new participants, which was often rate-limited by outreach budgets. The final challenge was data-driven program-wide adoption of successful initiatives; contributing factors included resource constraints, complex multistakeholder decision-making within a large consortium, downstream impact, and openness to effecting change based on impact evaluations.

**Figure 1:**
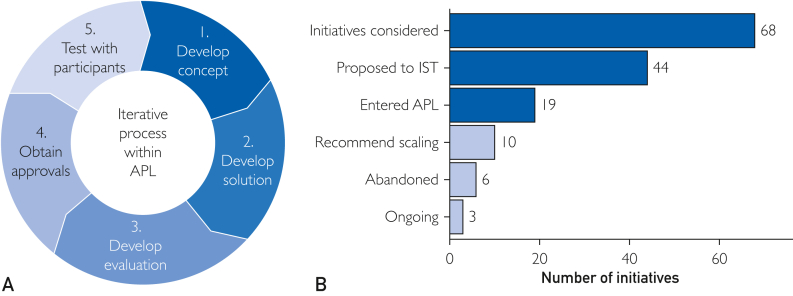
APL process and funnel. (A) Steps within APL. (B) Number of initiatives at different phases of entering and exiting APL; light blue bars show the current status of initiatives that entered APL. IST stands for Innovation Stewardship Team, a group of NIH and Scripps leadership who determined entry and exit for each initiative. APL, All of Us participant laboratories.

Each APL initiative was designed to address a systematic program challenge and then vetted to ensure it was a promising solution, with an appropriate cost–benefit and risk profile (Supplemental Table 3, available online at https://www.mcpdigitalhealth.org/). Within APL, each project went through an iterative process (Figure 1A). Numerous initiatives were considered, proposed to the program leadership committee, and approved (Figure 1B). Portfolio balancing and alignment with changing program goals were used (Supplemental Table 3). Despite APL’s stated goal to pursue high-risk, high-reward projects, small, low-risk projects were most likely to be approved. Six initiatives were abandoned once it became clear they were unlikely to meet their success criteria: 3 were abandoned due to operational constraints that arose before they were deployed to participants and 3 were abandoned based on results; of those 2 based on user testing results and 1 based on results with participants.

**Figure 2:**
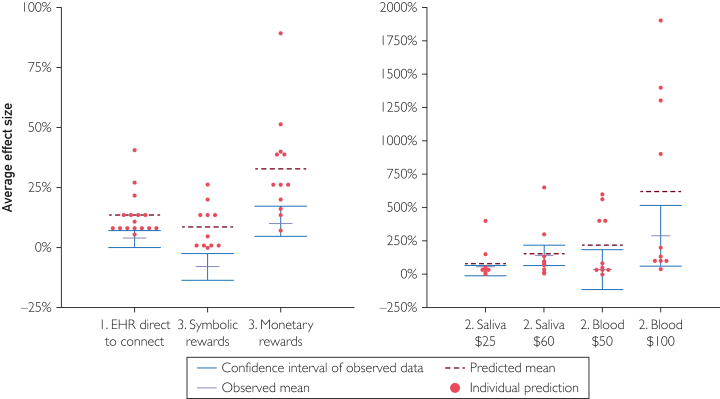
Prediction survey results with NIH and Scripps staff. Predictions for each initiative are shown in red, and results from randomized controlled trials are shown in blue. Staff consistently overestimated the effect size of initiatives, and the degree of overestimation varied.

On average, staff predicted that program outcomes would improve under 92% (n=11) of the initiatives that were launched to participants; in practice, 75% (n=9) improved outcomes. Two-thirds (n=115) of predictions were outside the 95% CI of the observed effect, although accuracy varied across initiatives: the most accurately predicted project had 53% of predictions within the CI, and for 1 initiative, 0% were within the CI. On average, predictions were more than double (104%) the observed effect (Figure 2).

### Increasing Task Completion

#### Streamlining Participant-Mediated EHR Sharing

##### Problem

The EHR connection module becomes available after participants complete EHR consent. Of eligible participants, 76% start the module, and 37% share an EHR.

##### Initiative

Instead of asking participants to navigate to the connection module from the dashboard, we transitioned directly to the connection module after EHR consent. This sought to increase the connection rate by reducing friction^12^ and providing clarity about next steps. We aimed to increase EHR connections by at least 3.5 percentage points.

##### Evaluation

We performed an RCT where participants either went to the connection module (n=5895) or to the dashboard (n=7401). Owing to technical constraints, we used a toggle approach where we switched between versions every 2 to 3 days. Our findings were robust to cohort effects (Supplemental Figure 3, available online at https://www.mcpdigitalhealth.org/).

##### Results

Sending participants directly to the connection module substantially increased the proportion of participants who selected their health care provider, from 75.7% to 91.1% (*P*<.001). However, the connection rate only increased by 3.5% (from 39.9% to 41.3%; *P*=.09), indicating considerable attrition in the connection module. We observed no differential effects across UBR categories.

##### Scaling

Although we fell short of our success criteria, EHR direct connection was implemented because it had a benefit at no cost; we also added trackers to enable identification of attrition within the sharing module. This intervention yields 20 additional EHRs for every 1000 participants who complete EHR consent.

#### Financial Compensation for Donating a Biospecimen

##### Problem

*All of Us* generates genomic data from blood samples, collected on site, and saliva samples, collected by mail. Among invited participants who proceed in a self-guided journey, 26% donate blood and 33% donate saliva. Participants who donate blood are offered $25 to offset time spent and travel expenses for onsite appointments. The program sought to increase compensation in response to inflationary pressure and participant feedback and to consider offering compensation to participants who donate saliva.

##### Initiative

We fielded a survey on perceived fairness of compensation amounts, followed by a 6-arm RCT to evaluate donation rates in response to different compensation amounts. The fairness survey (n=6000, 91% UBR overall and 72% UBR R/E) and qualitative user testing (n=15) informed the amounts tested in the RCT (Supplemental Figure 4, available online at https://www.mcpdigitalhealth.org/). For saliva, the RCT evaluated the program’s baseline of $0 against $25 and $60; for blood, it evaluated the program’s baseline of $25 against $50 and $100. Importantly, $200 for blood was the median response from the fairness survey but was not included in the RCT because it exceeded the program’s budget, and there were concerns about the potential for coercion. We approached new potential participants via the Amazon Mechanical Turk platform, and 7100 opted in to being given information about *All of Us,* including saliva and blood donation options and compensation available, along with a custom link to enroll. Of those who opted in, 68% were UBR overall and 34% were UBR R/E. A subset were invited for follow-up qualitative interviews (n=21) after the evaluation. Our success criterion was obtaining actionable data on compensation levels that are deemed fair and effective.

##### Results

Increasing compensation increased biospecimen donation. However, the impact varied substantially depending on the biospecimen type and the level of compensation (Table 1). For saliva, both compensation rates had a statistically significant effect on data donation rates (by 140% for $25 and by 320% for $60); for blood, offering $100 increased donations by 274%, but $50 had no effect. Offering $25 for saliva cost $45 per additional biospecimen (accounting for the cost of giving $25 to the participants who would have donated if offered $0), making it the most cost-effective compensated option. Offering $50 for blood was the least cost-effective because it did not meaningfully increase the biospecimen donation rate.

**Table 1:** Evaluation Groups for Biospecimen Compensation Evaluation and the Recorded Donation Rates

Biosample type	Compensation level ($)	Enrollment (%)	Biosample donation (%)^a^	Cost per additional biospecimen ($)
Saliva (n=1488)	0 (control)	6	0.5	0
Saliva (n=1528)	25	9∗∗	1.2∗	45
Saliva (n=1670)	60	10∗∗∗	2.1∗∗∗	81
Blood (n=856)	25 (control)	13	0.35	0
Blood (n=796)	50	10	0.39	407
Blood (n=762)	100	12	1.31∗	127

∗*P*<.05; ∗∗*P*<.01; ∗∗∗*P*<.001, relative to the baseline compensation for that biospecimen type. Regression outputs are in Supplemental Appendix 4 (available online at https://www.mcpdigitalhealth.org/).

a Both enrollment and biospecimen donation rates were calculated as intent to treat.

Qualitative interviews suggested that participants experienced more barriers to completing a blood donation compared with those for saliva donation, because of needing to find time for the appointment and travel to a physical site; this may explain why shifting the donation rate required more significant compensation. Separately, we found a positive effect of compensation on the completion of tasks beyond biospecimen donation. The spillover effect was strongest for the $100 blood group, increasing subsequent unrelated program data donation task completion by 167% (*P*=.015) compared with 45% to 67% for the other compensation levels (*P*>.1). We observed no differential effects across UBR categories. When asked in the qualitative interviews, no participants flagged concerns about coercion as a result of the compensation offered (Supplemental Tables 8-11).

##### Scaling

A key objective of the program is to collect samples from 1 million participants by the end of 2026. Extrapolating the evaluation findings, the program could exceed that target by offering $100 for blood and would get 94% of the way via the most economically efficient amount of $25 for saliva (Supplemental Tables 12 and 13). *All of Us* is considering these data because it decides whether to change compensation.

#### Symbolic Rewards and Financial Compensation for General Engagement

##### Problem

Although offering compensation holds promise to increase study participation, there is also research showing that nonfinancial incentives, such as status awards^13^ and gamification via points-based systems,^14^ can change behavior. Scaling these incentives is of low cost.

##### Initiative

We designed a user interface where participants could earn points for each task they completed, and a progress bar displayed their total points (Supplemental Figure 5, available online at https://www.mcpdigitalhealth.org/). At specific thresholds, participants would reach a new status, such as “Explorer” or “Champion” and see a celebratory image. In addition, we tested offering a $25 gift card to participants who completed all tasks.

##### Evaluation

The user experience was updated to the point-based interface for all participants (∼85,000). The first 2061 participants enrolling after launch had a 50% chance of being assigned to the experience with just points (n=1061) or the monetary add-on (n=1000). We then randomly selected a subset of participants who had enrolled before the evaluation launch date and had only completed the first survey module. Of this group, we randomly assigned a subset to the points (n=1017) and monetary groups (n=1013). Owing to technical constraints, we were not able to simultaneously evaluate the experience without points. Therefore, we created an analytical comparison group who enrolled at least 120 days before the start of the evaluation (n=39,435). We aimed to increase data donations for each of the groups by 20%.

##### Results

Compensation increased tasks completed by 17% for new participants (from 4.1 to 4.8 tasks; *P*<.001), whereas we recorded a small decrease for existing participants (from 0.2 to 0.1 tasks; *P*=.02) compared with the points system only (Table 2). Comparing the points-based system to the no points experience, we observed an 8% reduction in task completion (from 4.4 to 4.1 tasks; *P*=.005) for new participants. The net effect of both points and compensation was an 8% increase in task completion for new participants. For all outcomes, the direction and size of the effect were comparable across UBR categorizations (Supplemental Tables 14-19). Our conclusions continue to hold after bootstrapping the no points comparison cohort (Supplemental Tables 14-19).

**Table 2:** Evaluation Groups Symbolic Rewards and an Added Monetary Incentive Evaluation

Participant cohort	User experience	Completed tasks	Completing all tasks (%)
Before comparison group	No points, no compensation (n=39,435)^a^	4.4	4
Newly enrolled participants	Points only (n=1000)	4.1	4
	Points and $25 compensation (n=1061)	4.8∗∗∗	7∗∗
Previous enrolled participants	Points only (n=1013)	0.2	0.4
	Points and $25 compensation (n=1017)	0.1	0.2

∗*P*<.05; ∗∗*P*<.01; ∗∗∗*P*<.001, relative to the points group for the newly or previously enrolled participant cohort. Regression outputs are in Supplemental Appendix 5 (available online at https://www.mcpdigitalhealth.org/).

a This group was not randomly assigned. Instead, we created a before comparison group of participants who had enrolled at least 120 days before the start of the evaluation and looked at the tasks they completed over a 120-d period.

##### Scaling

Although monetary rewards cost approximately $18 per additional task, the negative effect of the points system led us to recommend against scaling. This highlighted the importance of testing individual components of incentive structures because they may have offsetting effects. The program could consider a similar monetary reward without a point system or consider task-specific compensation.

### Broadening Biospecimen Donation Accessibility

#### Expanding Biospecimen Collection Appointment Times

##### Problem

Program-collected blood samples are overnighted to the Biobank at Mayo Clinic. Collections that cannot arrive the next day should include only the 3 of 7 tubes with sufficient stability. As a result, biospecimen collections at many locations were restricted to weekdays before noon. Participants indicated that later weekday or weekend times were often preferable.

##### Initiative

We piloted expanding appointment times, including weekdays 1 to 6 pm, Saturdays 12 to 5 pm, and Sundays 7 am to 5 pm, at a local blood collection center. Participants who were not available at the standard appointment times were offered extended hours and informed that this would constitute a partial, rather than a full, blood collection, which may limit the number and type of results the program could return to them in the future.

##### Evaluation

Program staff offered collections at expanded times to: **(**1) 96 previously invited participants who could not make the standard appointment times and (2) 776 participants who were invited to donate a sample for the first time. Over the 9-week pilot, we aimed to add 45 collections, complete a full collection from at least 3 participants who had previously declined, and complete a partial collection from at least 7 participants who had previously declined.

##### Results

During the pilot, we recorded 182 collections, with 43 (24%) partial collections during expanded times; all success criteria were met (Table 3). Of the new participants who were invited to complete collections, 133 (73%) completed a full collection and 24 (13%) completed a partial collection. Of the 96 participants who previously declined to complete a collection, 6 (6%) completed a full collection and 19 (20%) a partial collection. It is noteworthy that in the cohort of previously approached participants, 6 (25%) were full rather than partial collections. Qualitative interviews with participants suggested that the information provided about the differences in collection type gave participants a better understanding of the need for restricted collection times and the limitations of samples collected during extended hours. There were no material changes to the program or enrollment processes during the pilot that would have encouraged participants who had previously declined biosample collection to reconsider. This suggests that the information is valuable for participants in deciding when and what type of donation they want to pursue.

**Table 3:** Expanded Collection Times Met All Success Criteria and Improved on UBR R/E Collection Rates at Scale, Compared With the Pilot, Likely Owing to the Demographic Characteristics at the Blood Collection Centers Where It Was Scaled

Success criteria	Goal	Actual	Status
Total collections	45	182	Exceeded
Previously invited participants (n=96)	10	25	Exceeded
Full collections	3	6	Exceeded
Partial collections	7	19	Exceeded
Newly enrolled participants (n=776)	NA	157	NA
Full collections	NA	133	NA
Partial collections	NA	24	NA
UBR R/E rates	Pilot (%)	Scale (%)	
Incremental collections at original times	21	28	
All expanded (h)	30	31	
Weekday expanded (h)	21	28	
Weekend expanded (h)	28	31	

UBR R/E, historically underrepresented in biomedical research based on race and/or ethnicity.

##### Scaling

The initiative was scaled to 4 additional program partners. Six months after the pilot, this initiative resulted in 81 incremental collections. Full collections, at original appointment times, from UBR R/E participants increased at scale, influenced by the preexisting larger UBR R/E populations at those blood collection centers.

#### Distributing Saliva Kits Through Community Partners

##### Problem

Community partner feedback indicated that adding in-person saliva kit pickup options to delivery by mail could improve biosample donation uptake.

##### Initiative

We aimed to determine the feasibility of distributing kits through community partners. Partners representing communities at 10 locations volunteered to participate and received 350 saliva kits. Participants near these partners could choose between receiving a kit in the mail and picking it up (Supplemental Figure 6, available online at https://www.mcpdigitalhealth.org/).

##### Evaluation

We sought to distribute at least 200 kits through community partners, including 75% of kits to UBR participants and 50% of kits to UBR R/E participants. We aimed to have 75% of distributed kits returned to Biobank by mail, in line with rates for kits that participants receive by mail.

##### Results

Goals for kit return and distribution to UBR participants exceeded; however, the goal for kit distribution was not (Table 4). Debriefs with community partners revealed confusion about eligibility criteria and some partners operating on the outdated information that DNA results would not be available to participants who donate saliva.

**Table 4:** Community Partner Saliva Kit Distribution Goals and Results

Success criteria	Goal (%)	Actual (%)	Actual (n)	Status
Kit return rate	75%	81%	129	Met
UBR overall	75%	97%	125	Met
UBR R/E rates	50%	88%	114	Met
Kit distribution rate	57%	37%	194	Not met

UBR R/E, historically underrepresented in biomedical research based on race and/or ethnicity.

##### Scaling

The program scaled this initiative to any interested community partner and its mobile tours (traveling exhibits that feature information about the program as well as opportunities to participate),^15^ along with updated training materials to address partner feedback. As of July 2024, 32 partners across 20 states had ordered kits to distribute.

## Discussion

**Figure 3:**
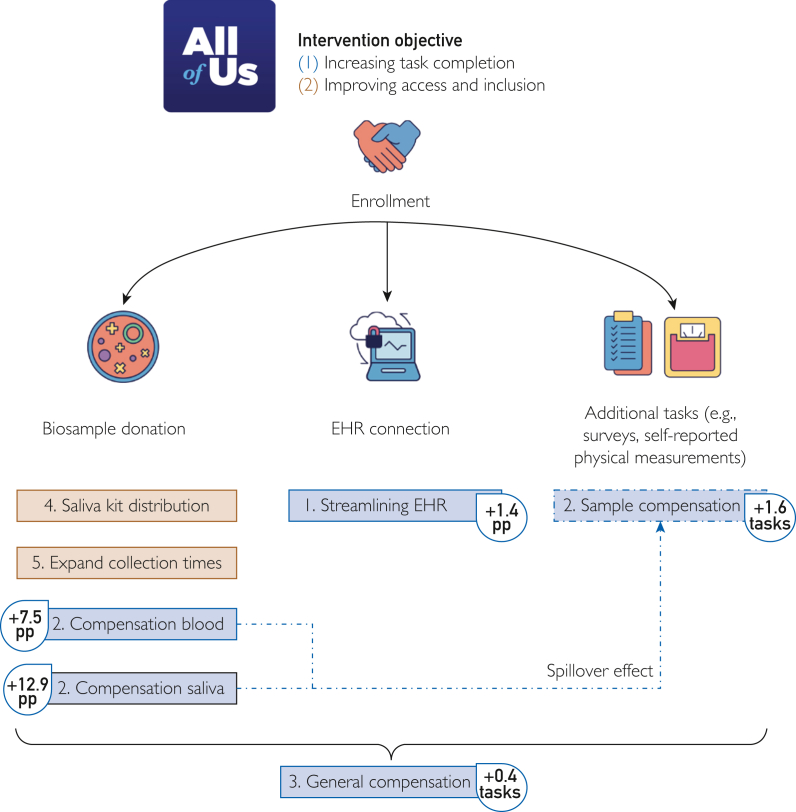
Cases have a positive impact across the participant journey, from consent completion through retention. Compensation cases had additional effects on task completion beyond the compensated tasks.

We developed and evaluated processes to rapidly test strategies not only aiming to improve program engagement and retention but also minimizing risk and disruption to the broader program. We created scalable, data-driven strategies to help the program optimize resource allocation and reach its goals. By focusing on initiatives that could improve accessibility and compensate participants for their time, as well as evaluating all efforts for differential effects on UBR participants, we improved recruitment and retention across the participant journey (Figure 3). Collaboration with the IRB and use of an umbrella protocol–streamlined processes.

Prediction survey results show that, on average, staff overestimated the effect of interventions by more than double, in line with other literature.^16^^,^^17^ Importantly, all initiatives that failed to positively affect program outcomes were abandoned, although 92% of initiatives were predicted by staff to be effective a priori; this underscores the importance of building evidence to inform scaling decisions and ensuring that evaluations are well-powered. As of this writing, the program scaled 50% of recommended initiatives. While this exceeds the implementation rate of communication interventions at government institutions,^18^ scaling is not necessarily straightforward owing to limited resources and difficulty reallocating resources.

A limitation of our approach is that some initiatives could not be evaluated in a way that sufficiently addressed all key confounds, such as expanding biosample collection times, which was not a randomized evaluation. Additional studies should be conducted to validate the conclusions drawn from these evaluations. Further, although the estimated causal effect in the evaluation is accurate, the results may, nonetheless, fail to manifest at scale because of deployment differences that the original evaluation did not account for,^18^ such as current events at the time of enrollment, changes to recruitment campaigns, and lack of implementation fidelity.^19^ Although we do not believe that this takes away from the importance of innovation development and testing as described in this article, it underscores the benefits of measuring impact as initiatives graduate from successful evaluation to deployment at scale.^19^^,^^20^ Finally, findings from specific initiatives may not generalize to research programs with different participant characteristics and engagement and retention challenges.

Nonetheless, we believe some learnings are likely to apply more broadly, including streamlining tasks and improving accessibility to increase UBR participation, offering compensation to achieve larger effects on behavior compared with user experience changes, and positive spillovers of compensation to unrelated program tasks. We continue to monitor initiatives when they are scaled and recommend that others use a similar approach, whether scaling their own findings or those from other studies.

## Conclusion

We found that a large, federally funded program with hundreds of thousands of participants can create a dedicated testing environment to de-risk innovation activities and recommend successful initiatives for scaling; we recommend that similar studies adopt a learning cohort model. Further, we identified tactics to improve health research engagement and retention, with a focus on UBR overall and UBR R/E communities, which can be used by other health research studies.

## Potential Competing Interests

Dr Topol reports NIH grant NIH UM1TR004407 and consulting fees from Tempus Scientific Advisory Board, Abridge Health Scientific Advisory Board, and Pheno.ai. Dr Kheterpal is an employee and shareholder of CareEvolution. The other authors report no competing interests.

## Ethics Statement

The human subjects’ research described in this manuscript was reviewed and approved by the *All of Us* Research Program institutional review board. Consent to participate in the *All of Us* Research Program was documented for all program participants.
