# Supplementary material for: A Model for Rapid Innovation for Engagement, Enrollment, and Data and Sample Collection in a Diverse Cohort Study: Insights from All of Us Participant Labs
Source: Mayo Clin Proc Digit Health. 2025 May 21;3(3):100227. doi: 10.1016/j.mcpdig.2025.100227 (PMC12179602; doi:10.1016/j.mcpdig.2025.100227)
Supplement: Supplementary Material [file mmc1.docx]

# Supplemental Materials

A model for rapid innovation for engagement, enrollment, data and sample collection in a diverse cohort study: Insights from *All of Us* Participant Labs

[**Supplemental Materials 1**](#_ziaakywsi73y)

[APL Initiative Selection and Abandonment 1](#_a1mci3tgeol6)

[Evaluating APL Processes 3](#_khhi03yug3zj)

[Supplemental Tables 5](#_kqm9ltqm06x4)

[All of Us Participant Labs: Proposal Review Form 9](#_kbcszqccu225)

[Initiatives: Additional Context 12](#_8n3e4fempwxk)

[S1. Streamlining EHR sharing 12](#_bcpzsfqo9pej)

[Regression outputs 13](#_j6qjm65sfb5h)

[S2. Financial compensation for donating a biospecimen 14](#_554r2mbx7zz7)

[Biosample donation qualitative interviews 15](#_x2b8lh2ru0t8)

[Qualitative interview plan 16](#_rxro38f5wwan)

[Regression outputs 20](#_qhn1gr3aagxc)

[S3. Symbolic rewards and financial compensation for general study engagement 23](#_xmp8suvamn6)

[Regression outputs 24](#_gmt97da34tle)

[Robustness check 26](#_opczjeix1id7)

[S4. Expanding biospecimen collection appointment times 27](#_4l6l453uorqm)

[S5. Community partner kit distribution 28](#_l8rbuwoj08ev)

## APL Initiative Selection and Abandonment

APL had four operational components: 1) setting innovation priorities, 2) creating dedicated innovation workstreams, 3) establishing research compliance processes, and 4) encouraging high risk initiatives and failing fast. Inspired by industry best practices^1^, we created a dedicated innovation workstream to ensure that time and effort were focused on identifying and implementing concepts to test. *All of Us*’s standard processes included thorough vetting from numerous stakeholders in an effort to ensure that public-facing content was inclusive, culturally competent, and scientifically sound. We created an innovation workstream with streamlined versions of the same components, to shorten the time from idea to implementation while meeting due diligence requirements. For example, innovation efforts prior to the creation of APL often took multiple years to go from concept to implementation, and we sought to decrease that to less than six months. To avoid delaying program-wide efforts, we utilized dedicated innovation teams and technical resources. The size of the research program enabled us to assess improvements to the participant experience by exposing only a subset of research participants to a new approach, which helped reduce the risk to the overall program and enabled a process of learning and adaptation before efforts were scaled or abandoned.

Each idea went through a multi-step vetting process before being implemented. Potential initiatives were identified by methods including dedicated large and small group ideation sessions, proposals from program partners, and identifying solutions to specific problems as they arose. Ideas came from across the consortium, including NIH staff, TPC staff, and community partners. A customized scoring rubric was designed to determine whether a project met the program’s innovation goals (Supplemental Table 2). The scorecard was first used at TPC, where any member of the team was welcome to present their idea at a monthly “shark tank” style meeting along with a one-page summary. After discussion, all members of the team were invited to use the rubric to score ideas and provide feedback. Based on the scores and comments, TPC’s director identified which ideas would move onto the brief development phase. Using an agreed-upon template (see supplemental materials), TPC staff would complete briefs for the Innovation Stewardship Team’s (IST’s) review. IST was made up of NIH leadership and TPC’s director, and it determined the entry and exit of each initiative. Briefs were reviewed and scored at monthly IST meetings, and all IST members' scores were weighted equally. In the event of a borderline score, the decision was approved by the program’s CEO to enter APL. Once ideas entered APL, they were within the protected space for innovation. For research compliance purposes, an umbrella protocol was developed and approved by the program’s IRB, and each initiative was an appendix to that umbrella protocol. Once the umbrella innovation goals and guardrails were agreed upon with the IRB, individual project review was streamlined.

First, concept development included reviewing literature and relevant program data to ensure we invested in initiatives that were promising and untested. Next, landscape and competitive analyses were used to evaluate reach, cost, and quality, including build versus buy considerations. Throughout, we kept program goals at the forefront, including UBR focus, inclusivity, and research data quality. Second, we considered multiple approaches to the initiative. In many cases, we conducted interviews with current and potential users, asking a series of open-ended questions to understand their behaviors, needs, goals, motivations, preferences, and pain points. We considered development tradeoffs but did not let them dominate explorations. We utilized user testing to gauge the intuitiveness and effectiveness of potential solutions, identify problems, and observe users. We sought feedback from TPC’s Virtual Advisory Team (VAT), a group of 22 *All of Us* participants (77% UBR R/E). Third, we designed initiative-specific evaluations to describe whether the initiative met its success criteria and to support data-driven decision-making. In many cases, these were well-powered Randomized Controlled Trials (RCTs), which generally have high internal validity due to the use of random assignment and by using similar or identical outcome measures to those that would be used at scale^2,3^. We created pre-analysis plans with outcomes of interest, analytical frameworks, the number of experimental groups, and the target sample size^4,5^ to remove flexibility in analytical approach after data collection^6–8^. RCTs were powered for effect sizes in the 1-3 percentage point range, close to what has been found in other settings^9^. Depending on ethical, logistical, or financial constraints, we also sometimes elected to utilize quasi-experimental or retrospective analyses to assess the causal impact of an initiative without random assignment^10,11^. Feasibility testing generally did not utilize RCTs. Before launch, stakeholders were often asked to make predictions about how the initiative would perform, which helped benchmark findings and increase accountability and learning^12,13^.

Finally, establishing a culture of innovation, with multiple process points where abandoning efforts was considered, was crucial to APL’s success. It was agreed that no topic was off limits–even if it had been off limits in prior discussions–and every idea would be thoughtfully considered. This was crucial to create an environment with free, expansive thinking.

After data collection and analysis, if a project met its success criteria, it was presented to IST for consideration to exit APL, using a scoring rubric that mirrored the entry criteria (Supplemental Table 3). If approved to exit APL, the project was outside the innovation infrastructure and was under consideration for scaling through the consortium-wide process that considered costs and benefits in the context of program strategic goals, resources, staffing, and budgets.

Further, it was important to dedicate time to identifying new initiatives and to allow individuals to structure that time based on their workstyle: some preferred group brainstorming meetings and others preferred independent thinking followed by collecting feedback. By reviewing proposals monthly, a pipeline of initiatives was created and iterated upon. Just as important was creating the physiological safety for an initiative to fail fast. Individuals often become attached to their work and can perceive a project’s failure as their own, and a panoply of systemic biases incline people to continue escalating commitment to a project even in the face of negative evidence^14^. To avoid this, we discussed the rationale each time a project was abandoned, including a cost-benefit analysis and opportunity costs, and we explicitly celebrated the individual who made the difficult choice to abandon a project.

## Evaluating APL Processes

*All of Us* Participant Labs began as a pilot in February 2022 and was evaluated in March 2023. Goals were set for rapid review timelines and the number of projects approved to enter and exit APL. To establish and maintain this independent innovation workstream, it was crucial to have support and commitment from program leadership across departments. Leadership communicated innovation as a program priority, made decisions that supported innovation when resource constraints arose, and committed to monthly meetings to discuss APL. We strove to establish department-specific time commitments to innovation (e.g., 10% of each department’s time); although we were not successful at implementing this, we recommend it to those considering establishing an innovation infrastructure. Rapid review timelines for IST were requested to ensure concepts were quickly evaluated before significant resources were invested in initiatives. One crucial component to this was including the Director of Health Equity as an IST member to ensure that each initiative was evaluated by IST from an Engagement and Health Equity perspective. Further, IST provided support to maintain APL’s dedicated innovation workstream as well as guidance and resources for specific initiatives. The pilot was able to meet all goals; however, the risk of meeting the goal for implementation time frame was increased for projects with more regulatory complexity.

One challenge that delayed some projects was the program’s Institutional Review Board (IRB) prioritizing reviews of mainline work and deliberations regarding which initiatives required full board review. There were multiple initiatives that could have been implemented without an evaluation and would not have needed IRB review on their own; however, because we were evaluating the effects of the changes, they required review. To rectify this, we collaborated more closely with research compliance staff. In an ideal scenario, we would recommend identifying IRB staff who specialize in timely review of innovation initiatives. On average, initiatives were expected to launch within 4 months of entering APL but actually launched about 11 months after entering APL. We surpassed the goals for a number of projects to enter and exit APL. We also streamlined the NIH information security review processes for these initiatives. This was thoughtfully done to ensure we were reaching the threshold the program set to feel comfortable offering something as part of the research program, while avoiding labor intensive review processes.

Following the evaluation of APL in March 2023, the program decided to continue APL with the recommendation to focus on disruptive initiatives. IST defined disruptive as “introducing a component of the participant experience that is fundamentally different from the activities the program has previously conducted.” A disruptive initiative had the potential to make a substantial contribution to at least one of the following program goals: 1) Enrollment: increase enrollment and/or core participation, in particular amongst UBR R/E participants, by at least 2,500 participants per year or scalably decrease cost per acquisition by at least $50; 2) Retention: increase retention, in particular amongst UBR R/E participants, by at least 2%; 3) Data collection and ancillary studies: includes the collection of at least one new data/biospecimen type, data/biospecimen collection, or participant engagement method with high scientific value to the program (see Figure S1 below); 4) Return of value: return unique value to participants, that they could not otherwise access without significant cost (opportunity, financial, or risk).

At this time, we also evaluated the distribution of project scores to see if there was a clear difference between those that did and did not enter APL. Since the cutoff score of 20 was near a local maximum, and there was a local minimum at 21, we moved the cutoff to 20.6 (Figure S2). We maintained the practice that IST may allow for judgment calls to be made to overturn the result that is produced based on the score.


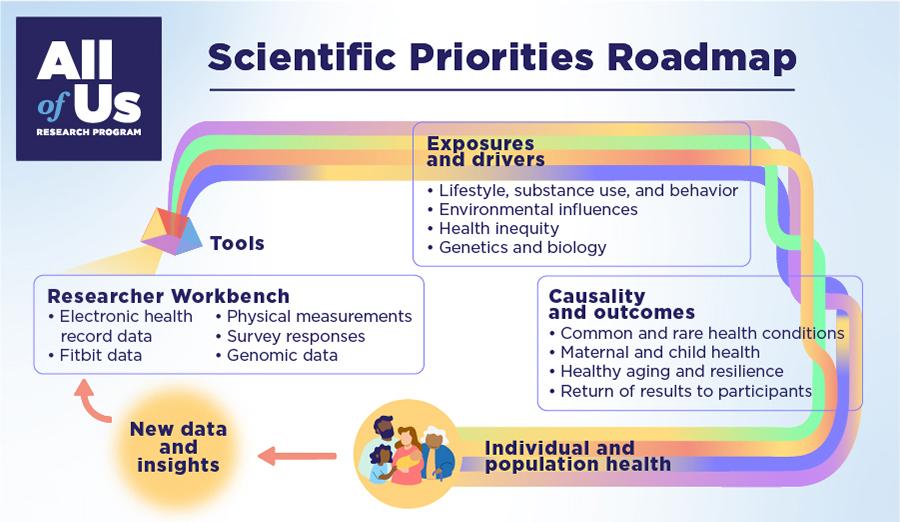
Supplemental Figure 1: The All of Us Research Program’s Scientific Roadmap.


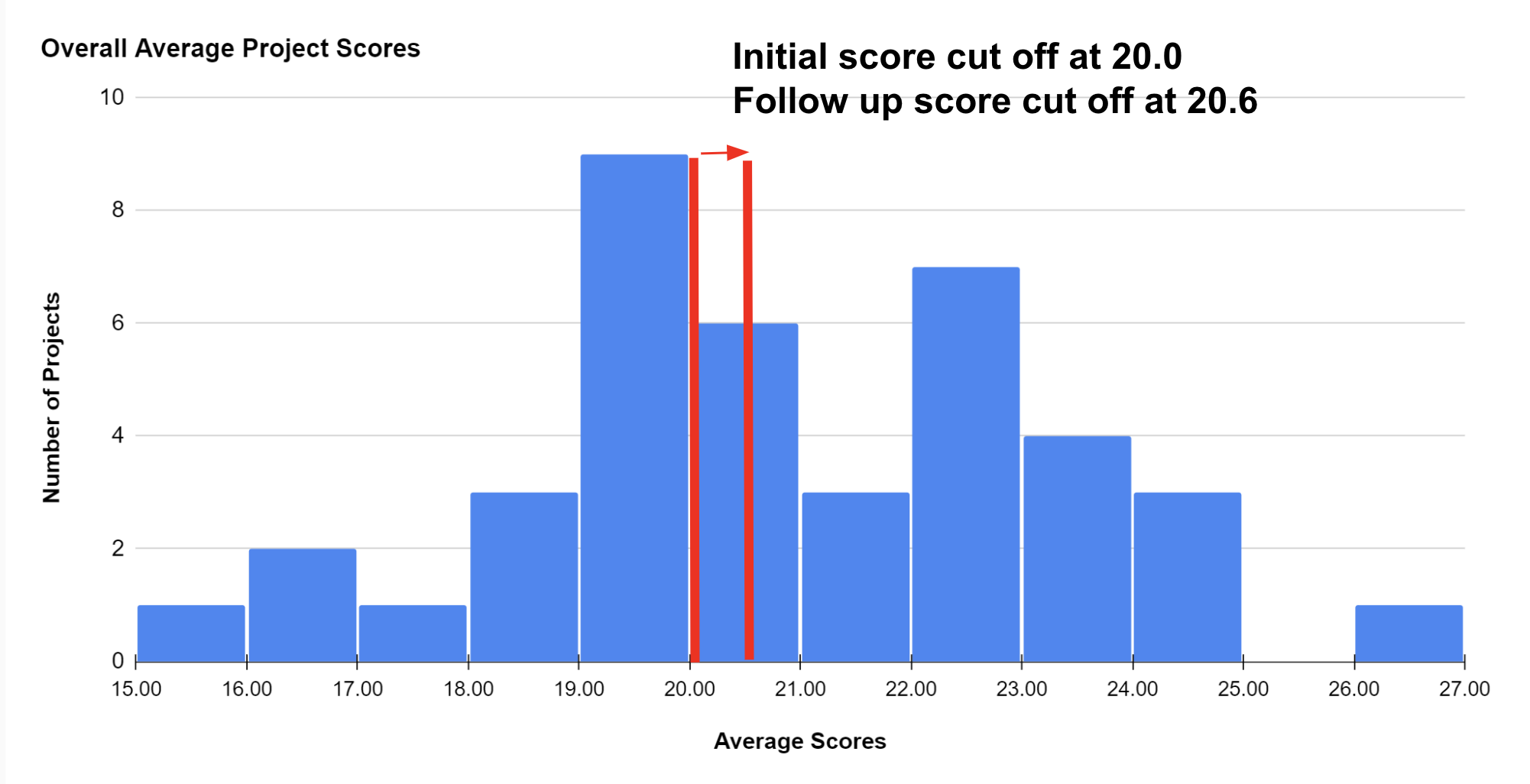


Supplemental Figure 2: Changing the APL Entry Cutoff

## Supplemental Tables

| **CRITERIA** | **Lowest Score (0)** | **Highest Score (10)** | **Comments** |
| --- | --- | --- | --- |
| **OVERALL STRATEGIC ALIGNMENT** | | | |
| Introduces a component of the participant experience that is fundamentally different from the activities the program has previously conducted | No novel change to program activities | Paradigm shift for the program |  |
| Will impact UBR communities positively and help the program reach UBR goals and advance health equity | Unhelpful to UBR goals | Will help reach UBR goals |  |
| Degree of alignment to program goals: enrollment, engagement, retention, data collection, ancillary studies, and/or return of value | Lack of alignment to any program goals | Considerably aligned with any program goals |  |
| **Strategic Alignment Score (0 to 10)** | | | <Enter Strategic Alignment Score Here> |
| **POTENTIAL IMPACT AT SCALE** | | | |
| Potential to meet at least one of these metrics at scale:   - Increase enrollment, in particular amongst UBR R/E participants, by at least 2500 participants per year and/or scalably decrease cost per acquisition by at least $50; - Increase retention by at least 2%, in particular amongst UBR R/E participants; - Provides at least one new data/biospecimen type, data/biospecimen collection, and/or participant engagement method with high scientific value to the program that aligns with the scientific roadmap); or - Returns unique value to participants, that they could not otherwise access without significant cost (opportunity, financial, and/or risk) at scale | Does not have potential to meet at least one metric at scale | Very significant contribution to one or multiple metrics at scale |  |
| Has the potential to help the program reduce costs at scale | No potential to reduce costs at scale | Huge cost savings for the program can be realized at scale |  |
| Will positively impact all current and new participants (ie. “participant market”) at scale | Will impact small or niche market with no or limited market growth at scale | Will impact very significant market size with high market growth at scale |  |
| **Potential Impact Score (0 to 10)** | | | <Enter Potential Impact Score Here> |
| **PILOT FEASIBILITY & FISCAL RESPONSIBILITY** | | | |
| Degree of pilot complexity (technical, policy, IRB, communications, engagement, support center, etc.) relative to pilot value | Complexity is too high relative to pilot value | Complexity is in proportion to pilot value |  |
| Degree of financial investment needed to run pilot relative to pilot value | Very high program budget investment relative to little pilot value | No or limited program budget investment required relative to high pilot value |  |
| Length of expected time to results for pilot | Very long investment period relative to pilot results | Short time to pilot results |  |
| Secondarily, consider potential cost **at scale** relative to value and whether it could be offset by public-private partnerships | High cost per participant without clear opportunities to offset cost | Low cost per participant and/or potential partnerships can offset costs |  |
| **Feasibility & Fiscal Responsibility Score (0 to 10)** | | | <Enter Feasibility & Fiscal Responsibility Score Here> |
| **TOTAL SCORE**  **30 total possible points; minimum score of 20 needed for pass consideration** | | | **<Enter Total Score Here>** |

***Note****: We recognize that each project is unique and may have considerations that are not adequately captured by this scorecard. Therefore, the IST may allow for judgment calls to be made to overturn the result that is produced based on the score.*

Supplemental Table 1. APL entry criteria

| **CRITERIA** | **Lowest Score (0)** | **Highest Score (10)** | **Comments** |
| --- | --- | --- | --- |
| **STRATEGIC ALIGNMENT** | | | |
| Alignment with current program needs, including inclusion of UBR communities. Have they changed? | Not aligned with program needs | Aligned with program needs |  |
| Did the project meet project-specific success criteria defined by project champion & TPC? | Did not meet success criteria | Project exceeded success criteria |  |
| **Strategic Alignment Score (0 to 10)** | | | <Enter Strategic Alignment Score Here> |
| **POTENTIAL IMPACT** | | | |
| What is the expected ROI at scale, particularly for UBR communities? | Would have minimal impact on the program’s goals relative to effort required | Would have significant impact on at least one program goal relative to effort required |  |
| Do the results of the impact evaluation allow us to evaluate how generalizable the results are? | Impact evaluation was underpowered and/or biased | Impact evaluation was well powered and representative of the *All of Us* experience |  |
| **Potential Impact Score (0 to 10)** | | | <Enter Potential Impact Score Here> |
| **FEASIBILITY & FISCAL RESPONSIBILITY** | | | |
| How difficult will this be to implement at scale (includes cost, technical, policy, IRB, communications, engagement, security, etc. requirements)? If the difficulty is high, is this justified by the expected return? Consider impact on and potential barriers for UBR communities. | Difficulty is too high relative to expected return | Difficulty is proportional or less than expected return |  |
| Were any significant risks discovered? | Significant risks discovered | No or insignificant risks discovered |  |
| **Feasibility & Fiscal Responsibility Score (0 to 10)** | | | <Enter Feasibility & Fiscal Responsibility Score Here> |
| **TOTAL SCORE**  **30 total possible points; minimum 20 points needed for pass consideration; minimum 15 to consider re-testing.** | | | **<Enter Total Score Here>** |

***Note****: We recognize that each project is unique and may have considerations that are not adequately captured by this scorecard. Therefore, the IST may allow for judgment calls to be made to overturn the result that is produced based on the score.*

Supplemental Table 2. APL exit criteria.

| **Primary Program Goal** | Approved | Proposed to IST | % approved |
| --- | --- | --- | --- |
| Enroll & retain 1M participants | 8 | 21 | 38% |
| Expand data available | 8 | 17 | 47% |
| Return value to participants | 3 | 6 | 50% |
|  |  |  |  |
| **Initiative Size** |  |  |  |
| Small | 5 | 7 | 71% |
| Medium | 10 | 22 | 45% |
| Large | 4 | 15 | 27% |
|  |  |  |  |
| **Initiative Risk** |  |  |  |
| Low | 13 | 21 | 62% |
| Medium | 4 | 18 | 22% |
| High | 2 | 5 | 40% |
|  |  |  |  |
| **Initiative Potential** |  |  |  |
| Incremental | 12 | 18 | 67% |
| Breakthrough | 5 | 18 | 28% |
| Disruptive | 2 | 8 | 25% |

**Supplemental Table 3**. APL Portfolio Summary

## *All of Us* Participant Labs: Proposal Review Form

**Project Name:**

**Bottom Line Up Front:** In a few short sentences, summarize the project and its major impacts if it were to be successful at scale; call out alignment to program goal(s): enrollment, engagement, retention), data collection, ancillary studies, and/or return of value.

1. **Project brief**

Description (Background, Study Design, Budget Estimate)

- Make sure to include: Degree of pilot complexity, number of participants, clear success metrics with power calculations, degree of financial investment of the project, and length of expected time to see results for pilot

What problem does this initiative aim to solve?

- Make sure to include: How your project Introduces a component of the participant experience that is fundamentally different from the activities the program has previously conducted

What does success look like? Quantify this if at all possible

What are the UBR goals?

- Make sure to include: How your project will impact UBR communities positively and how it will help the program reach UBR goals and advance health equity; quantify UBR inclusion goals if at all possible.

What do we expect to learn?

What are the early stopping rules? Consider any unhelpful impact on UBR communities.

If successful, what would be the impact if we were to scale?

- Estimate financial impact of scaling (this does not need to be precise - an order of magnitude investment, with notes about how it scales - e.g. noting one time costs vs per participant costs will be sufficient)

Which of the below metrics does the project have the potential to meet?

1. Increase enrollment, in particular amongst UBR R/E participants, by at least 2500 participants per year and/or scalably decrease cost per acquisition by at least $50;
2. Increase retention by at least 2%, in particular amongst UBR R/E participants;
3. Provides at least one new data/biospecimen type, data/biospecimen collection, and/or participant engagement method with high scientific value to the program that aligns with the [scientific roadmap](https://docs.google.com/document/d/1QwUsDm7aCdGAgFawGtiTJqGxEq44tZ4-/edit#heading=h.kpikvur20p47)); or
4. Returns unique value to participants, that they could not otherwise access without significant cost (opportunity, financial, and/or risk) at scale

1. **Is the APL project aligned with at least one of the program’s goals? If so, bold the relevant one(s) and change the color to green.**

Program Goals

- Enroll 1M participants who reflect the diversity of the US, cover the lifespan, and have shared all baseline elements. Of these participants, 500,000 are actively engaged.
- Expand data available for 1M participants to include surveys, health data streams, WGS, environmental data, and physical measurements.
- Launch ancillary studies as a core and scalable capability, expanding the cohort and delivering phenotypic, lifestyle, environmental, and biological data.
- Establish a diverse global community of at least 10,000 researchers productively using *All of Us* data.
- Incorporate participant return of value into data collections and assess its impact, including return of information to participants on genomics and EHR.

If you selected at least one, please explain which objective and how it aligns in a sentence or two. If you selected more than one, indicate which is primary.


1. **Bold in green which groups this project will include:**

- *All of Us* participants
- External participants
- Both *All of Us* and external participants
- No participants

1. **Implementation timeframe: How do you expect it to take to implement the project? (Bold in green your selection.)**

- <2 months
- 2 months
- 4 months
- 6 months
- > 6 months

1. **Evaluation timeframe: How long do you expect it to take to evaluate the project? (Bold in green your selection.)**

- <2 months
- 2 months
- 4 months
- 6 months
- > 6 months

1. **Is this a small/medium/large-scale initiative? (Bold in green your selection.)**

- Small (target 1 month for NIH review with no IRB review required, e.g. A/B testing messaging)
- Medium (target 2 months for NIH and possible IRB review, e.g. A/B test pre-consent, download a PDF of health history based on PPI responses)
- Large (target 3 months for NIH, IRB, policy/security review, e.g. at-home physical measurements pilot, participant community)

1. **What is the risk for this project? (Bold in green your selection.)**

- Low (30% chance of success)
- Medium (15% chance of success)
- High (5% chance of success)

1. **Has an NIH project champion been proposed for this project? (Bold in green your selection.)**

- Yes
- No

If the response is “yes”, please provide the name of the project champion:


1. **Review:**

- This proposal was reviewed on ___________
- The decision was to (move ahead, revise, stop) ____________

## Initiatives: Additional Context

### S1. Streamlining EHR sharing

Internal user testing revealed several pain points to connecting EHR, including privacy concerns, lack of perceived benefit, and confusion about how to share EHRs. Quantitative analysis showed that 76% of those who provided consent started the connection module and 40% eventually ended up connecting a record.


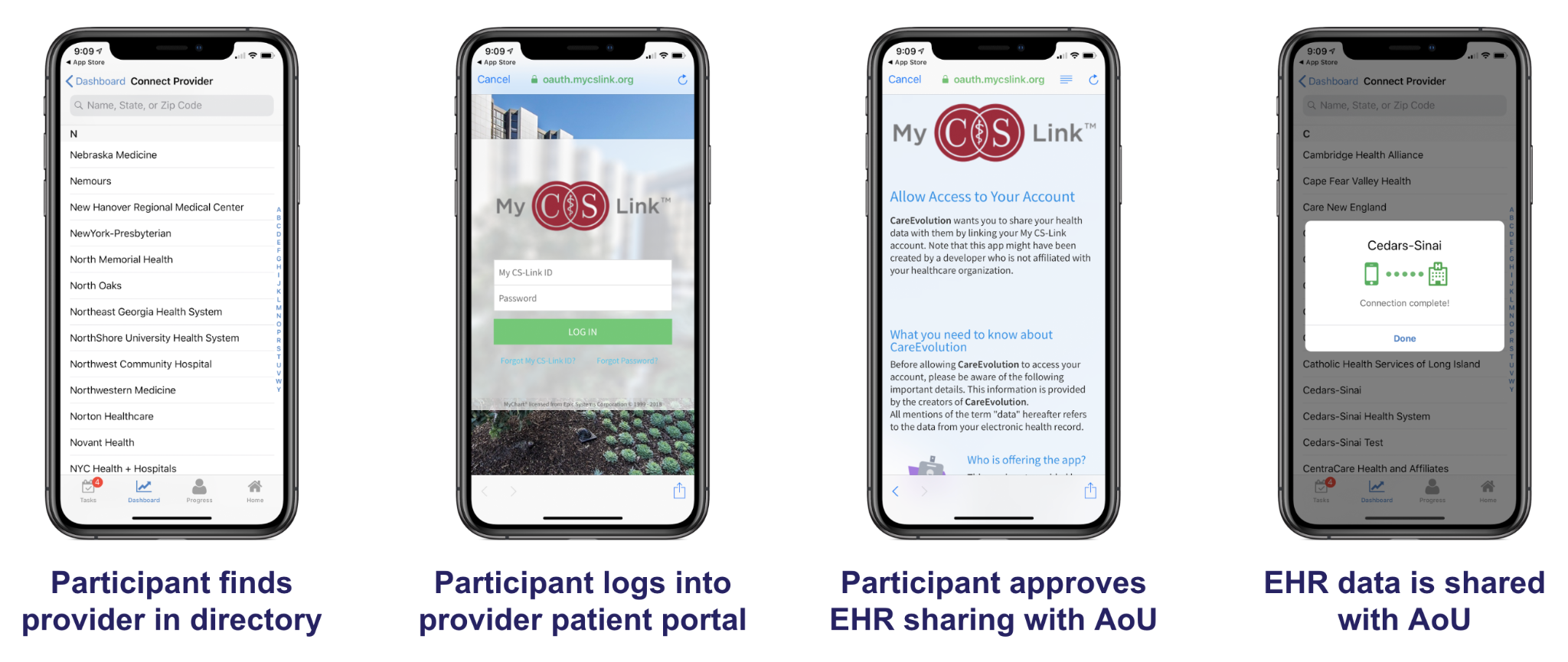


Supplemental Figure 3. Participant-Mediated EHR sharing process, using Cedars Sinai as an example. Note that *All of Us* never accesses the participant’s login information.

The toggle approach yields results close to random sampling if, in a given time period, participant characteristics (both known and unknown) are similar and that there are no external events that somehow impact enrollment behavior (e.g., a widely publicized study security breach). We believed that such an assumption was reasonable because the time period was short (several days compared to several weeks). Importantly, participant demographics and retention metrics differed depending on the recruitment campaign, but traffic from specific campaigns was typically spread out over a period of 1-2 weeks. Nonetheless, during the evaluation we encountered one event where enrollment spiked over several days, which led to unbalanced randomization. Despite this, our findings were robust to cohort effects. The experimental treatment (i.e., directing participants to the connection module after completing EHR consent) became statistically significant (p<0.01) when [demographic controls](#slide=id.g1eac14a45d7_0_22) were added to the regression and weakly significant (p=0.06) when we [controlled for the surge period](#slide=id.g1fe7ab4840f_3_14) that contributed to an unbalanced sample. This increased our confidence that we were observing a genuine impact of the treatment rather than a spurious result.

Participants who successfully connected an EHR made an average of 1.4 connections.

##### Regression outputs

|  | **% Connection module start (provider search screen)** | **EHR Connection rate %** |
| --- | --- | --- |
| Streamlining | 1.192***  (0.053) | 0.055  (0.036) |
| Constant (no streamlining) | 1.137***  (0.027) | -0.409***  (0.024) |
| N | 13,296 | 13,296 |
| Pseudo R-squared | 0.041 | 0.000 |
| *** p<0.001; ** p<0.01; *p<0.05. | P < 0.001 | p = 0.121 |

Table S4: Outputs from Logistic regression capturing the effect of starting EHR connection and completing EHR connection based on EHR consent streamlining

|  | **% Connection module start (provider search screen)** | | | **EHR Connection rate %** | | |
| --- | --- | --- | --- | --- | --- | --- |
|  | **UBR participants** | **UBR r/e participants** | **RBR participants** | **UBR participants** | **UBR r/e participants** | **RBR participants** |
| Streamlining | 1.238***  (0.067) | 1.427***  (0.107) | 1.109***  (0.089) | 0.062  (0.042) | 0.053  (0.073) | 0.032  (0.067) |
| Constant (no streamlining) | 1.245***  (0.033) | 0.956***  (0.053) | 0.899***  (0.047) | -0.371***  (0.028) | -0.631***  (0.050) | -0.499***  (0.044) |
| N | 9,461 | 3,284 | 3,835 | 9,461 | 3,284 | 3,835 |
| Pseudo R-squared | 0.049 | 0.066 | 0.042 | 0.000 | 0.000 | 0.000 |
| *** p<0.001; ** p<0.01; *p<0.05. | P < 0.001 | P < 0.001 | P < 0.001 | p = 0.139 | p = 0.468 | p = 0.635 |

Table S5: Outputs from Logistic regression capturing the effect of starting EHR connection and completing EHR connection based on EHR consent streamlining for UBR cohorts

|  | **EHR Connection rate %** | **EHR Connection rate %** |
| --- | --- | --- |
| Streamlining | 0.098** (0.036) | 0.069 (0.036) |
| Constant (no streamlining) | -1.429*** (0.108) | -0.453*** (0.031) |
| Demographic controls | Yes | NA |
| Surge controls | NA | Yes |
| N | 13,296 | 13,296 |
| Pseudo R-squared | 0.000 | 0.000 |
| *** p<0.001; ** p<0.01; *p<0.05. | p = 0.007 | p = 0.055 |

Table S6: Outputs from Logistic regression capturing the effect of completing EHR connection based on EHR consent streamlining with demographics and surge period controls. Demographic controls include participant demographics on UBR, age, education, income, birthsex, gender, and sexual orientation as regression variables. Surge controls denotes participants who enrolled to AoU during a significant enrollment surge via CVS campaign in December 2022 as a regression variable

### S2. Financial compensation for donating a biospecimen

By collecting biospecimens, *All of Us* enables researchers to make discoveries that would otherwise be impossible. Blood samples can be collected at a participating blood center or by ordering a collection kit and taking this to a Quest location or health care provider organization in their area. For simplicity, we framed this as “at your doctor” in our survey. Once participants become eligible to donate a biosample, they are prompted to choose one of these options. Users who enrolled received a reminder about the compensation available to them if they donated a biosample, if any, and were issued a gift card code via email upon completion.

Initiative success criteria also included: 1) Quantitative data on compensation amounts for core *All of Us* activities that are perceived as fair and that would motivate individuals to complete these tasks, 2) Quantitative data on subgroup differences for UBR overall and UBR R/E individuals, 3) Quantitative data on differences in stated compensation levels before and after the individual has experienced the task in question, which was crucial for retention efforts.

Prior to the RCT, we explored perceptions of fairness and self-reported compensation requirements in a large-scale survey of 6,000 diverse respondents. Respondents were randomized to survey arms asking about saliva donation, Quest blood donation, or blood donation at their doctor’s office. The median fair amounts for saliva, Quest, and one's doctor were $60, $100, and $100 respectively, while the median personally motivating amounts were all $100. All amounts were considerably higher than the current program offerings of $0 for saliva and $25 for blood. Figure S4 below displays the spread of answers below $250, which comprised 4,729 out of 6,000 responses.


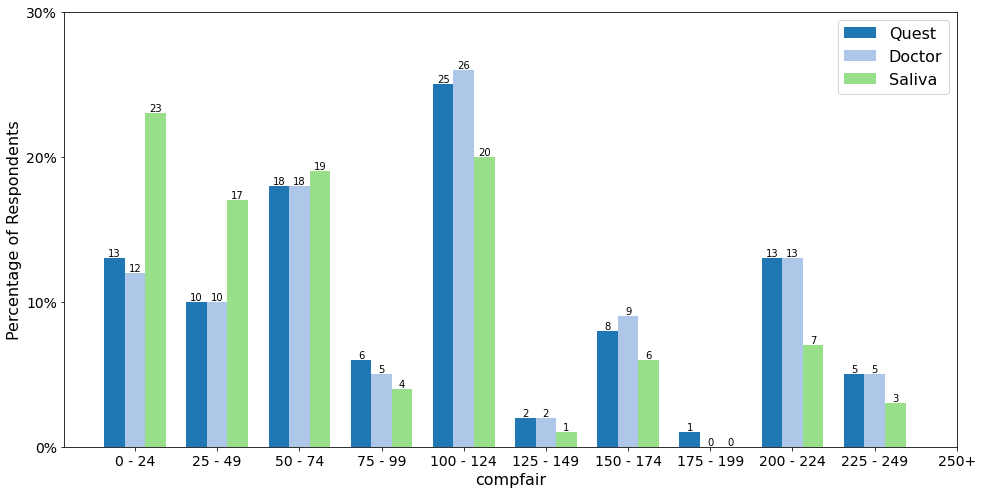


Supplemental Figure 4. Requested compensation amounts for biospecimen donation from the fairness evaluation.

The fairness evaluation also had a qualitative component consisting of 15 semi-structured interviews with non-*All of Us* users. Users were recruited and interviewed remotely via UserZoom, an industry-grade UX research platform. They were screened to ensure they had no familiarity with *All of Us* and so to meet UBR representation targets. For each of the three arms, we asked five users about compensation and fairness. Key themes included an expectation that users would receive compensation and a sense that $25 was too little for the value of their time and DNA information. Users were positively motivated by altruism but demotivated by mistrust and concerns about sharing sensitive health data.

##### Biosample donation qualitative interviews

We conducted qualitative interviews with a subset of participants (N=21) after data collection was completed to understand their experiences. Participants that enrolled in *All of Us* as part of the evaluation (n=1,599) received information about the opportunity of a 30 minute interview and could express their interest. We invited a subset of those interested to ensure coverage across different treatment arms, biosample donation completion behavior, and UBR r/e representation. The breakdown of the sample is given in table S7. Participants that completed an interview received $30 in the form of an Amazon or Target gift card.

| **Treatment arm** | **Total interviews** | **UBR r/e** | **Donation behavior** |
| --- | --- | --- | --- |
| Saliva, $0 | 3 | 1 | 2 donated, 1 not |
| Saliva, $25 | 4 | 0 | 2 donated, 2 not |
| Saliva, $60 | 4 | 0 | 2 donated, 2 not |
| Blood, $25 | 3 | 1 | 1 donated, 2 not |
| Blood, $50 | 3 | 2 | 1 donated, 2 not |
| Blood, $100 | 4 | 3 | 2 donated, 2 not |

Table S7: Breakdown of the sample for the qualitative interviews

The interviews with participants in the evaluation suggested that they experienced significantly more barriers to completing a blood donation compared to saliva, mostly due to needing to find time for the appointment and traveling to a physical site. This aligned with self-guided participant data of the median completion time for a saliva sample (12.5 days) compared to an on-site blood donation (26.5 days). Compensation of $100 may have motivated some additional participants to overcome these barriers, whereas $25 and $50 did not. We found a positive effect of compensation on the completion of other data donation tasks beyond the biosample donation track (from 1.89 tasks for baseline compensation to 2.98 tasks for additional compensation, p<0.001). Directionally, the spillover effect was strongest for the $100 blood group, increasing subsequent task completion by 167% (p = 0.001) compared to 45–67% for the other compensation levels (p>0.1).

##### Qualitative interview plan

Objectives

- To observe what users perceive to be fair compensation and understand their rationale behind choosing certain compensation amounts.
- To understand users’ previous experiences with research participation and biosample donation and how that informs their perceptions of fair compensation.
- To understand differences in stated compensation levels before and after the individual has experienced the task in question, which is crucial for retention efforts.
- To determine the threshold between fair compensation and coercion.

Participants

A cohort of users separate from those in the quantitative evaluation will be screened, recruited, and appropriately compensated via UserZoom. We will recruit a sample of users who reflect the program demographics and are non-*All of Us* participants (e.g. 2 UBR R/E, 2 UBR overall, and 1 RBR per user segment).

User profile:

- Not familiar with All of Us
- UBR R/E: At least 6 users must identify as other than White and non-Hispanic
- UBR age/location: At least 6 users must be over the age of 65 and/or reside in a rural/non-metropolitan area (these UBR criteria are relevant barriers in terms of task burden. E.g an older person or person in a rural area may find it difficult to travel to a Quest appointment)
- At most 3 users must not fit any of the above criteria

Methodology

We will conduct 1 hour-long remote semi-structured interviews that focus on biosample donation and the user journey up to it, with at least 5 users assigned to saliva, on-site blood, and via a medical provider until data saturation has been reached (3 segments, n=15).

The user will be given information about a biosample donation task and the necessary prerequisites they would have to complete (primary consent, Basics, EHR and gROR consent), before they declare a compensation amount. They will then go through their assigned biosample donation module as a mock participant before declaring an updated compensation amount. They will also be asked a series of questions based around other insights we hope to learn. Throughout this process, users will be asked to “think aloud” so the interview moderator can understand their thought process behind the decisions they make, and the moderator can probe deeper as needed.

Within the quantitative evaluation, we will also include a few free-text questions where users can provide qualitative feedback on specific compensation amounts we are considering for biosample donation. Suggested framing for these questions as follows:

1. You indicated that $X would fairly compensate a participant for donating a biosample. Why is $X fair?
2. Currently, the program pays participants $25 for donating a biosample.
   1. Is $25 fair compensation for this task?
   2. Would you donate a biosample for $25?
3. Let’s say the program changes compensation from $25 to $X.
   1. Is $X fair compensation for this task?
   2. Would you donate a biosample for $X?

Interview Script

**Introduction**

Hello! Thank you for taking the time to participate in this user research study for the All of Us Research Program. My name is [name]; I’m a User Researcher working for All of Us and I’ll be interviewing you today. Before we begin, I’d like to provide some context for this study and go over some logistics with you.

All of Us is a nationwide health research program under the National Institutes of Health (NIH). Participants that enroll in the program are asked to complete several tasks, including completing surveys about their health, sharing their health records, and donating biosamples, such as a blood sample. Researchers will use this data to learn how our biology, lifestyle, and environment affect health, which may one day help them find ways to treat and prevent disease. The program seeks to build one of the most diverse health databases in history, and emphasizes enrolling people from communities who have historically been underrepresented in health research. Those who join the program will be contributing to an effort to reduce health disparities and improve the health of generations to come, and may learn about their own health along the way. We value our participants’ contributions, so we are conducting this study to better understand how we can fairly compensate them for their participation.

During this interview, I will be asking you to think from the perspective of a prospective All of Us participant. Please note that you are not actually being entered into All of Us, and you will not be asked to share your actual data.

This interview should take around an hour. Please answer my questions to the best of your ability, and please let me know if you are uncomfortable at any point. You are free to stop participating at any time. I will now start recording this session.

**Background**

I’m going to start off by asking some questions so that I can learn more about your background.

1. Where do you live? (Town, state, rural or urban, etc). What is your occupation?
2. Have you participated in medical research before? Would you ever consider participating in medical research?
3. What experience do you have with providing biosamples (blood, saliva, etc)? For example, have you provided a blood sample at a doctor’s office before? How did you feel about completing those tasks?
4. Have you ever been asked to donate a biosample for research/philanthropic purposes before? (e.g blood drive)
   1. If yes, did you choose to donate? Why or why not?
      1. What incentives were you offered, if any? How did that inform your decision? Would you donate again, if asked?
   2. If not, would you ever consider donating a biosample? What circumstances might inform that decision?

**Pre Task**

*User will be assigned to either at-home saliva, Quest, or via medical doctor. Information about tasks will be written on slides and presented to the user.*

Now, I’m going to share my screen. I am sharing descriptions of some tasks that a participant would be asked to complete during and after joining All of Us. Each task is optional, but needs to be completed to proceed to the next task. I will read this aloud and you can follow along. After this, I will be asking you what you think is fair compensation for completing these tasks. It’s important you understand what these tasks are, so take as long as you need to take in this information, and let me know if I can clarify anything.

*Information on screen (present in sequential order). The user will only get information about one biosample.*

1. To summarize, you had to complete three consent forms and answer questions about you, your work and your home life. You then request a biosample donation kit/schedule a biosample appointment and you would provide the biosample. (*total times: 45-50 mins for Quest, 35-40 mins for Saliva, 60 mins for PTSC).* What would be a fair monetary payment for the program to offer someone to complete these tasks?
   1. Why?
      1. What factors should the program take into consideration when deciding on a fair payment?
      2. Should all participants be paid this amount, regardless of location, age, etc?
   2. Currently, the program offers $25 to complete this donation. How do you feel about that?
   3. Do you prefer cash payment or gift cards? If you prefer gift cards, what kinds?
   4. Would any forms of non-monetary compensation be fair for someone to complete this task? Please explain.
2. How much do you feel you yourself would need to be paid to complete these tasks?
   1. Why?
      1. For PTSC: How far away from you is your medical doctor? How does that distance affect the amount you would want to be paid?
      2. For Quest: how far would you be willing to travel to a Quest location? How does distance affect payment?
   2. Would you consider any forms of non-monetary compensation to complete this task? Please explain.
   3. How valuable would you find the results of your biosample donation, such as learning about your ancestry? (This is similar to Ancestry.com or 23&me.) Were you aware of those benefits? Please explain.

**Task (for users on saliva and Quest arms)**

*Test accounts have been set up, with prerequisite steps already completed.*

I am going to send you a link to the All of Us portal, and provide you with the login information. Note that this is only a test account, so you are not being registered as an actual participant and you will not be donating any biosamples. After you have logged in, please share your screen.

Now, click on the “Share Biosamples” module on the top of the screen. At the end of this first screen, please select [assigned] sample. Go through the next few steps, following all the instructions, and please verbalize your thought processes and reactions to what you see. Once you reach the “Are you ready?” screen, do not click Submit.

**Post Task**

1. *For Saliva/Quest:* Now that you’ve gone through the module, I’m going to ask you the same questions I did before about fair payment for completing this task. You may update your responses or answer the same as you did before. [repeat questions 2-3, probing into why they did/didn’t change their answers].
2. *For Saliva/Quest*: I told you to pick [saliva/blood]. If you had the choice, which sample would you choose to donate? Would a different compensation amount be fair for [sample they weren’t assigned]?
3. *For Quest:* you selected a Quest location to donate blood. Would you have preferred to instead go to your doctor’s office for a blood donation? Would a different compensation amount be fair if you were going to a more familiar location?
4. *For your doctor:* you scheduled your appointment over the phone with me. Would you have preferred to schedule it yourself via a digital scheduling tool?
5. At what point do you feel like the compensation being offered might pressure participants into completing the task, even if they are not comfortable doing so?
   1. What would be an acceptable compensation range that is fair but does not pressure participants?

That wraps up this interview! Thank you so much again for participating. Any questions/concerns before we wrap up?

#####

##### Regression outputs

|  | **Saliva sample** | | |
| --- | --- | --- | --- |
|  | **Enrollment %** | **Biosample donation %** | **Number of additional tasks (exploratory outcome)** |
| Saliva, $25 compensation | 0.421** (0.133) | 0.843* (0.406) | 0.993* (0.413) |
| Saliva, $60 compensation | 0.503*** (0.132) | 1.368*** (0.380) | 1.163** (0.406) |
| Constant ($0 for saliva) | -2.692*** (0.100) | -5.218*** (0.334) | 2.110*** (0.297) |
| N | 4,686 | 4,686 | 441 |
| (Pseudo) R-squared | 0.003 | 0.001 | 0.021 |
| *** p<0.001; ** p<0.01; *p<0.05. | $25: p = 0.002  $60: p < 0.001 | $25: p = 0.038  $60: p < 0.000 | $25: p = 0.017  $60: p = 0.004 |

Table S8: Outputs from Logistic regression capturing the effect of enrolling into *All of Us* and submitting a biosample based on compensation level. Additional Ordinary Least Squares regression on additional tasks completed based on compensation level

|  | **Saliva sample** | | | | | | | | |
| --- | --- | --- | --- | --- | --- | --- | --- | --- | --- |
|  | **Enrollment %** | | | **Biosample donation %** | | | **Number of additional tasks**  **(exploratory outcome)** | | |
|  | **UBR overall** | **UBR r/e** | **RBR** | **UBR overall** | **UBR r/e** | **RBR** | **UBR overall** | **UBR r/e** | **RBR** |
| Saliva, $25 compensation | 0.388*  (0.166) | 0.495*  (0.217) | 0.510*  (0.226) | 1.561*  (0.638) | 2.373*  (1.051) | 0.077  (0.492) | 1.041  (0.530) | 1.363*  (0.661) | 0.862  (0.673) |
| Saliva, $60 compensation | 0.493**  (0.164) | 0.580**  (0.213) | 0.543*  (0.225) | 1.792**  (0.628) | 2.106*  (1.062) | 1.136*  (0.492) | 0.777  (0.521) | 0.928  (0.648) | 1.798  (0.659) |
| Constant ($0 for saliva) | -2.730***  (0.126) | -2.535***  (0.166) | -2.622***  (0.166) | -5.896***  (0.578) | -6.273***  (1.001) | -4.554***  (0.410) | 2.174***  (0.402) | 1.775**  (0.508) | 2.044  (0.445) |
| N | 3,224 | 1,559 | 1,462 | 3,224 | 1,559 | 1,462 | 263 | 162 | 178 |
| (Pseudo) R-squared | 0.003 | 0.005 | 0.004 | 0.003 | 0.008 | 0.000 | 0.023 | 0.042 | 0.015 |
| *** p<0.001; ** p<0.01; *p<0.05. | $25:  p = 0.019  $60:  p = 0.003 | $25:  p = 0.023  $60:  p = 0.006 | $25:  p = 0.024  $60:  p = 0.016 | $25:  p = 0.014  $60:  p = 0.004 | $25:  p = 0.024  $60:  p = 0.047 | $25:  p = 0.900  $60:  p = 0.021 | $25:  p = 0.051  $60:  p = 0.137 | $25:  p = 0.041  $60:  p = 0.154 | $25:  p = 0.202  $60:  p = 0.007 |

Table S9: Outputs from Logistic regression capturing the effect of enrolling into *All of Us* and submitting a saliva sample based on compensation level for UBR cohorts. Additional Ordinary Least Squares regression on additional tasks completed based on compensation level for UBR cohorts

|  | **Blood sample** | | |
| --- | --- | --- | --- |
|  | **Enrollment %** | **Biosample donation %** | **Number of additional tasks**  **(exploratory outcome)** |
| Blood, $50 compensation | -0.256 (0.157) | 0.073  (0.818) | 0.413 (0.446) |
| Blood, $100 compensation | -0.038 (0.151) | 1.330  (0.660) | 1.497** (0.431) |
| Constant ($25 for blood) | -1.935*** (0.103) | -5.650***  (0.578) | 1.656*** (0.284) |
| N | 2,414 | 2,414 | 313 |
| (Pseudo) R-squared | 0.002 | 0.000 | 0.005 |
| *** p<0.001; ** p<0.01; *p<0.05. | $50:  p = 0.101  $100:  p = 0.802 | $50:  p = 0.929  $100:  p = 0.044 | $50:  p = 0.356  $100:  p = 0.001 |

Table S10: Outputs from Logistic regression capturing the effect of enrolling into *All of Us* and submitting a blood sample based on compensation level. Additional Ordinary Least Squares regression on additional tasks completed based on compensation level

|  | **Blood sample** | | | | | | | | |
| --- | --- | --- | --- | --- | --- | --- | --- | --- | --- |
|  | **Enrollment %** | | | **Biosample donation %** | | | **Number of additional tasks**  **(exploratory outcome)** | | |
|  | **UBR overall** | **UBR r/e** | **RBR** | **UBR overall** | **UBR r/e** | **RBR** | **UBR overall** | **UBR r/e** | **RBR** |
| Blood, $50 compensation | -0.271  (0.196) | -0.258 (0.237) | -0.186  (0.263) | 1.064  (1.156) | 0.010  (0.008) | -0.007  (0.006) | 0.306  (0.617) | 0.551 (0.723) | 0.507  (0.655) |
| Blood, $100 compensation | -0.046  (0.191) | -0.045  (0.232) | -0.004  (0.247) | 2.150*  (1.062) | 0.022*  (0.009) | 0.002  (0.006) | 1.477*  (0.600) | 2.012**  (0.704) | 1.449*  (0.022) |
| Constant ($25 for blood) | -2.002***  (0.132) | -1.669***  (0.161) | -1.826***  (0.164) | -6.301***  (1.001) | 0 (0.006) | 0.007  (0.004) | 1.785***  (0.418) | 1.500**  (0.490) | 1.524***  (0.380) |
| N | 1,629 | 864 | 785 | 1,629 | 864 | 785 | 181 | 128 | 132 |
| (Pseudo) R-squared | 0.002 | 0.002 | 0.001 | 0.001 | 0.005 | 0.003 | 0.002 | 0.009 | 0.008 |
| *** p<0.001; ** p<0.01; *p<0.05. | $50:  p = 0.167  $100:  p = 0.811 | $50:  p = 0.275  $100:  p = 0.847 | $50:  p = 0.478  $100:  p = 0.987 | $50:  p = 0.357  $100:  p = 0.043 | $50:  p = 0.230  $100:  p = 0.011 | $50:  p = 0.299  $100:  p = 0.783 | $50:  p = 0.620  $100:  p = 0.015 | $50:  p = 0.447  $100:  p = 0.005 | $50:  p = 0.440  $100:  p = 0.022 |

Table S1`: Outputs from Logistic regression capturing the effect of enrolling into *All of Us* and submitting a blood sample based on compensation level for UBR cohorts. Additional Ordinary Least Squares regression on additional tasks completed based on compensation level for UBR cohorts

**Biosample donation rate relative to biosample donation eligibility for each of the compensation level groups:**

|  | **Saliva sample** |
| --- | --- |
|  | **Biosample donation %** |
| Saliva, $25 compensation | 0.796 (0.511) |
| Saliva, $60 compensation | 1.337** (0.494) |
| Constant ($0 for saliva) | -0.847* (0.398) |
| N | 119 |
| (Pseudo) R-squared | 0.036 |
| *** p<0.001; ** p<0.01; *p<0.05. | $25: p = 0.119  $60: p = 0.007 |

Table S12: Outputs from Logistic regression capturing the effect of submitting a saliva sample based on compensation level for biosample eligible participants.

|  | **Blood sample** |
| --- | --- |
|  | **Biosample donation %** |
| Blood, $50 compensation | 0.380 (0.892) |
| Blood, $100 compensation | 1.013 (0.728) |
| Constant ($25 for blood) | -1.846** (0.621) |
| N | 71 |
| (Pseudo) R-squared | 0.005 |
| *** p<0.001; ** p<0.01; *p<0.05. | $50:  p = 0.671  $100:  p = 0.164 |

Table S13: Outputs from Logistic regression capturing the effect of submitting a blood sample based on compensation level for biosample eligible participants.

### S3. Symbolic rewards and financial compensation for general study engagement

Effective non-monetary rewards status awards^15^ and gamification, such as point-based systems, which have been found to increase student performance and physical activity in children and adolescents^16–18^

Previously enrolled participants were informed about the changes corresponding to their experimental group by email, SMS, or push notifications, depending on their notification preference. For the analysis, we analyzed the number of program tasks completed over 120 days after the participant was exposed to the experimental condition.

7% of new monetary participants completed enough tasks to earn a $25 gift card, compared to 4% of symbolic participants (p=0.008). Of participants who earned a monetary reward, 87% participants claimed their gift card, in line with rates we have observed for claiming biosample gift cards (78%). Given that 7% of monetary participants completed the user journey to earn a $25 gift card and that 87% of those participants claimed their gift card, rewards cost approximately $18 per additional task.

^
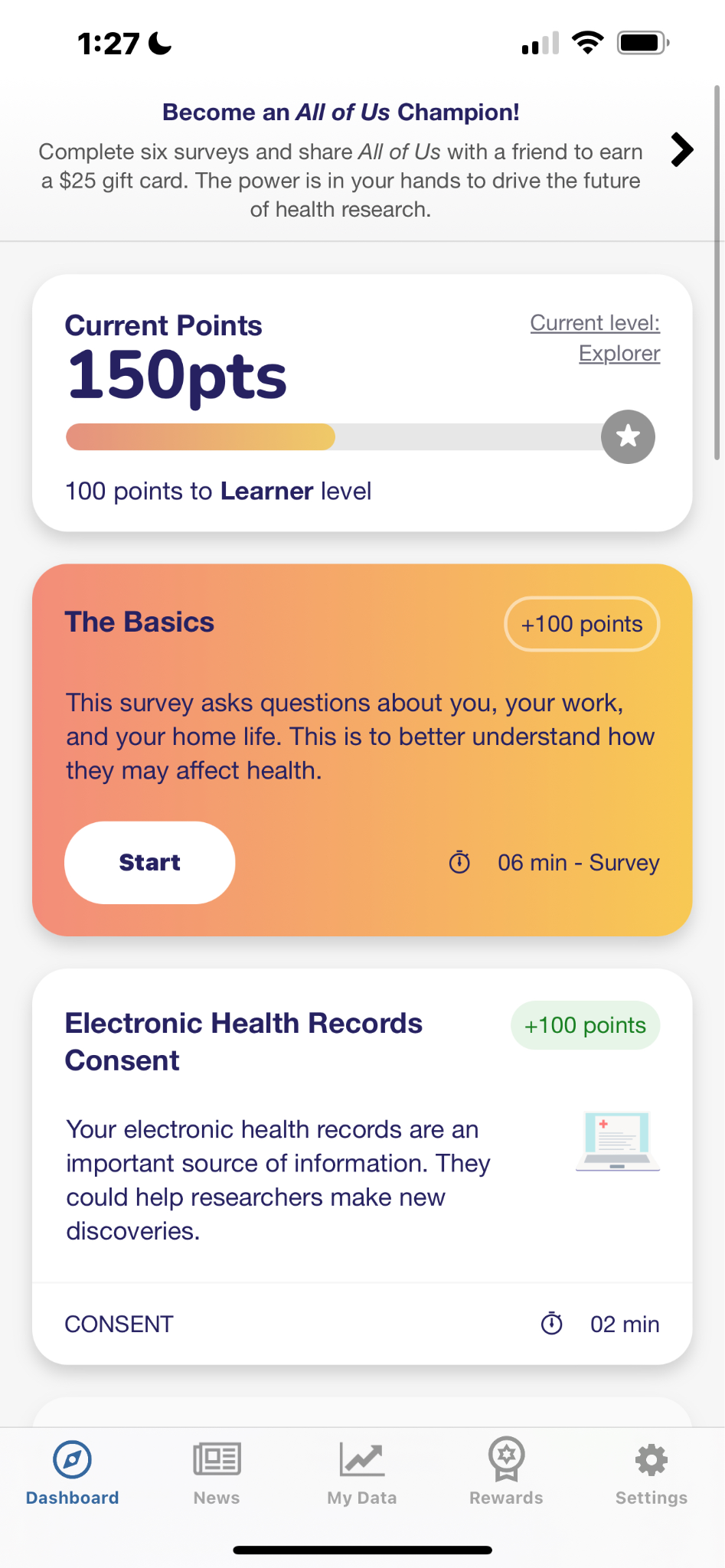

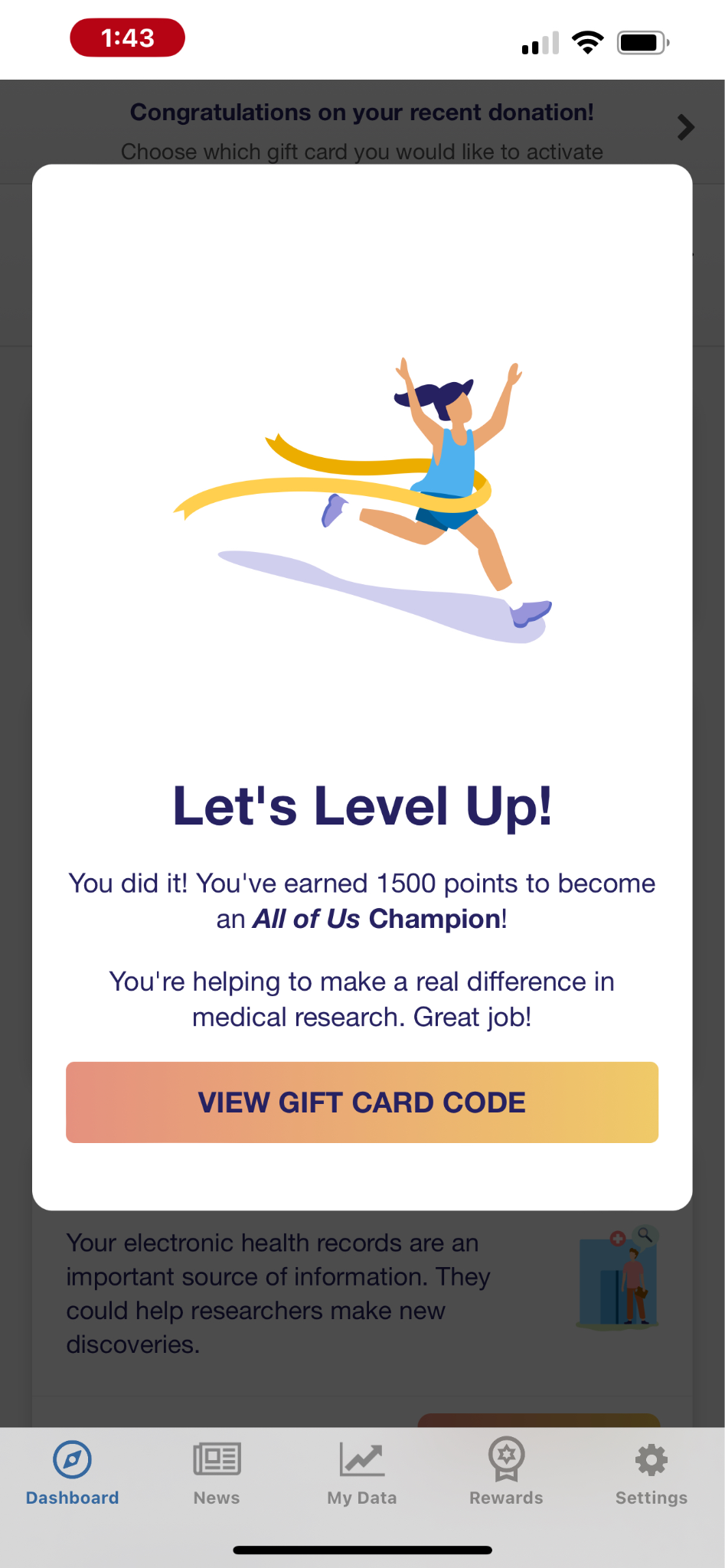

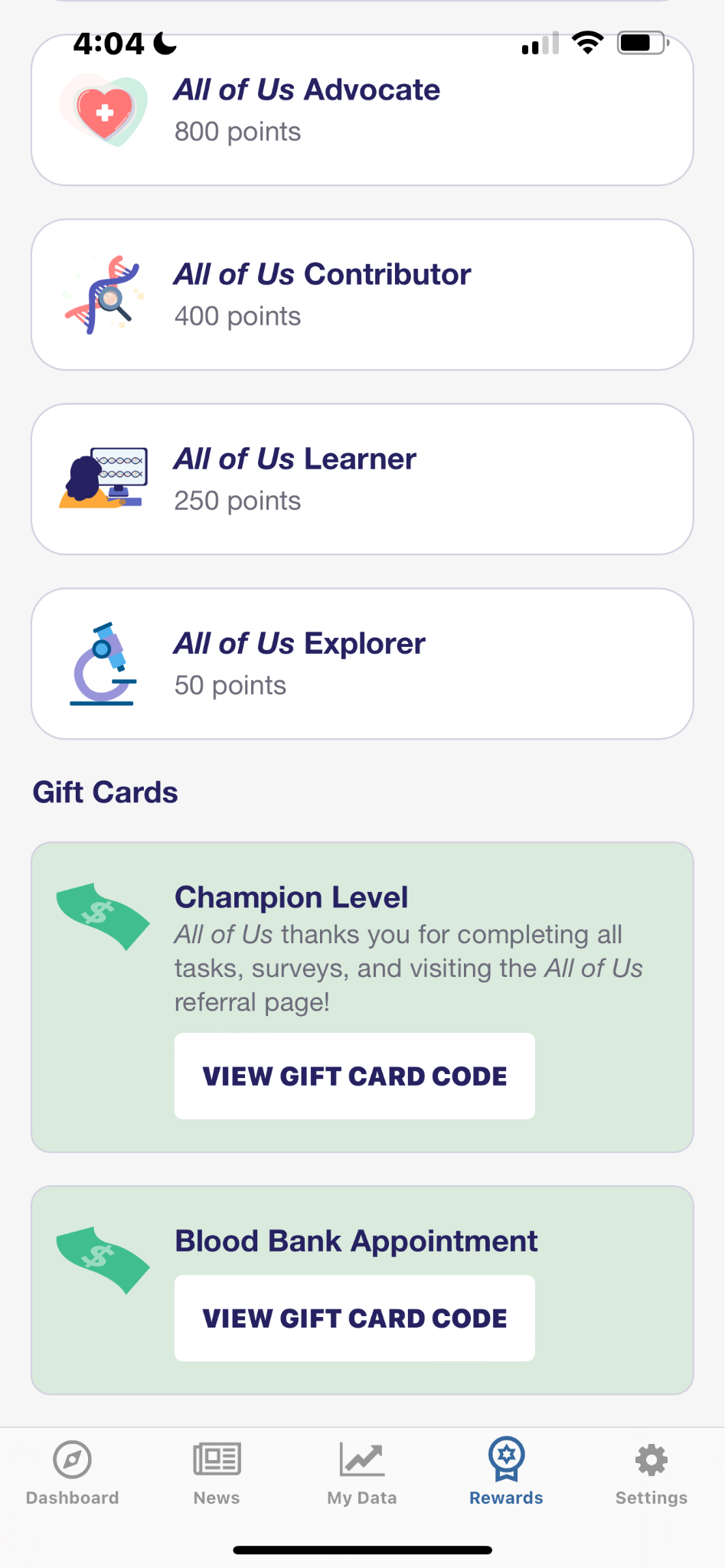
^

Supplemental Figure 5. Screenshots of the Rewards participant experience, showing the dashboard with a progress bar and points value (left), an example of a symbolic reward for achieving a new status (middle), and the achieved statuses along with available gift cards (right).

##### Regression outputs

|  | **Completed tasks** | | **% completing all tasks** | |
| --- | --- | --- | --- | --- |
|  | **Newly enrolled participants** | **Previously enrolled participants** | **Newly enrolled participants** | **Previously enrolled participants** |
| Points + money offered | 0.678*** (0.173) | -0.119* (0.052) | 0.531** (0.201) | -0.691 (0.867) |
| Constant (Points Only) | 4.099*** (0.121) | 0.259*** (0.036) | -3.164*** (0.156) | -5.534*** (0.501) |
| N | 2,061 | 2,030 | 2,061 | 2,030 |
| (Pseudo) R-squared | 0.007 | 0.003 | 0.008 | 0.008 |
| *** p<0.001; ** p<0.01; *p<0.05. | | | | |

Table S14: Outputs from Ordinary Least Squares regression capturing the number of tasks completed and Logistic regressions capturing the rate of all tasks completed based on exposure to points or points and compensation interfaces.

#####

|  | **Completed tasks** | **% completing all tasks** |
| --- | --- | --- |
| Points Only | -0.342** (0.121) | 0.0146 (0.158) |
| Constant (No points) | 4.441*** (0.020) | -3.179*** (0.026) |
| N | 40,496 | 40,496 |
| (Pseudo) R-squared | 0.000 | 0.000 |
| *** p<0.001; ** p<0.01; *p<0.05. |  |  |

Table S15: Outputs from Ordinary Least Squares regression capturing the number of tasks completed and Logistic regressions capturing the rate of all tasks completed based on exposure to points or no point interfaces

|  | **Completed tasks** | | | **% completing all tasks** | | |
| --- | --- | --- | --- | --- | --- | --- |
|  | **UBR overall** | **UBR r/e** | **RBR** | **UBR overall** | **UBR r/e** | **RBR** |
| Points + money offered | 0.148 (0.142) | 0.095 (0.206) | 0.306 (0.172) | 0.521* (0.223) | 0.612 (0.370) | 0.117 (0.382) |
| Constant (points only) | 2.576*** (0.101) | 2.392*** (0.142) | 1.479*** (0.118) | -3.726 (0.176) | -3.938*** (0.291) | -3.855*** (0.270) |
| N | 2,814 | 1,205 | 1,277 | 2,814 | 1,205 | 1,277 |
| (Pseudo) R-squared | 0.000 | 0.000 | 0.002 | 0.007 | 0.010 | 0.000 |
| *** p<0.001; ** p<0.01; *p<0.05. | | | | | | |

Table S16: Outputs from Ordinary Least Squares regression capturing the number of tasks completed and Logistic regressions capturing the rate of all tasks completed based on exposure to points or points and compensation interfaces by UBR cohort

|  | **Completed tasks** | | | **% completing all tasks** | | |
| --- | --- | --- | --- | --- | --- | --- |
|  | **UBR overall** | **UBR r/e** | **RBR** | **UBR overall** | **UBR r/e** | **RBR** |
| Points Only | -0.302* (0.145) | -0.239 (0.210) | -0.475** (0.176) | -0.158 (0.192) | -0.105 (0.312) | 0.445 (0.277) |
| Constant (No points) | 5.732*** (0.024) | 5.225*** (0.038) | 2.690*** (0.028) | -2.860*** (0.029) | -3.088*** (0.054) | -3.858*** (0.054) |
| N | 23,322 | 8,488 | 17,174 | 23,322 | 8,488 | 17,174 |
| (Pseudo) R-squared | 0.000 | 0.000 | 0.000 | 0.000 | 0.000 | 0.000 |
| *** p<0.001; ** p<0.01; *p<0.05. | | | | | | |

Table S17: Outputs from Ordinary Least Squares regression capturing the number of tasks completed and Logistic regressions capturing the rate of all tasks completed based on exposure to points or no point interfaces by UBR cohort

##### Robustness check

An important limitation of our evaluation is that we only randomized participants to either a points or points plus monetary compensation interface. To understand the effect of the points system on its own, we sought to compare the points group to participants that went through the All of Us program before the point evaluation was rolled out. Participants in this comparison group were not randomly assigned, which introduces possible confounds. Primarily, we are concerned about systematic differences between participants that were recruited through different recruitment campaigns at different times. Table S18 shows the composition of recruitment campaigns for the randomized cohort and the comparison group. While the same campaign sources occur in both groups, they do so in different proportions (e.g., 35.5% of participants in the comparison cohort were recruited via CVS campaigns compared to 7.6% of the randomized cohort).

|  | **Randomized cohort** | | **Comparison cohort** | |
| --- | --- | --- | --- | --- |
|  | **N** | **Proportion** | **N** | **Proportion** |
| **Total enrollment** | **2061** | **100%** | **39,435** | **100%** |
| **By campaign source** | | | | |
| Nextdoor | 899 | 43.62% | 14235 | 36.10% |
| CVS | 157 | 7.62% | 13984 | 35.46% |
| San Diego Blood Bank | 138 | 6.70% | 484 | 1.23% |
| Walgreens | 106 | 5.14% | 321 | 0.81% |
| Denver Health | 77 | 3.74% | 1144 | 2.90% |
| Mary’s Center | 68 | 3.30% | 290 | 0.74% |
| Participant referral | 64 | 3.11% | 318 | 0.81% |
| BloodWorks NorthWest | 59 | 2.86% | 89 | 0.23% |
| National Alliance of Hispanic Health | 38 | 1.84% | 205 | 0.52% |
| Fitbit | 25 | 1.21% | 54 | 0.14% |
| Google Play | 21 | 1.02% | 431 | 1.09% |
| WebMD | 14 | 0.68% | 909 | 2.31% |
| Sensis | 6 | 0.29% | 325 | 0.82% |
| Amazon | 6 | 0.29% | 452 | 1.15% |
| No source attributed | 331 | 16.06% | 5301 | 13.44% |
| Other | 52 | 2.53% | 893 | 2.27% |

Table S18: Breakdown of recruitment campaigns for participants enrolled as part of the evaluation and the comparison group.

We run a robustness check where we randomly sample 1,000 participants from the comparison cohort such that we maintain the same proportion of recruitment campaign sources as the randomized cohort. For example, in our randomized cohort 7.6% of participants enrolled through campaigns with CVS, so we ensure that 7.6% of the participants in our random sample for the comparison group were recruited via a CVS campaign. For this random sample of 1,000 participants, we calculate the average number of tasks completed within the first 120 days (our primary outcome measure). We then repeat this sampling and average task completed calculation 1,000 times and take the average across these iterations. This gives us an average number of tasks completed of 4.47 with a 95% confidence interval of 4.46 and 4.48 for the lower and upper bound, respectively. This average is relatively close to the average across the full cohort of 39,435 participants (4.44 tasks). Rerunning the OLS regression reported in Table S15 finds the same result as our original analysis, ie., a statistically significant decrease in task completion for the points-only cohort relative to the no-points comparison group (p=0.002). This regression output is reported in table S19.

While this gives us more confidence that the effect can be attributed to the interface changes rather than recruitment campaign differences, we cannot rule out all possible confounds, notably time-specific effects and small differences between campaigns run at different times within the same vendor and channel. Follow-up work should aim to address these limitations of our study.

|  | **Completed tasks** | **% completing all tasks** |
| --- | --- | --- |
| Points Only | -0.373 ** (0.119) | 0.0063 (0.006) |
| Constant (No points) | 4.472*** (0.085) | 0.0342*** (0.004) |
| N | 2,061 | 2,061 |
| (Pseudo) R-squared | 0.005 | 0.001 |
| *** p<0.001; ** p<0.01; *p<0.05. | | |

Table S19: Outputs from Ordinary Least Squares regression capturing the number of tasks completed and Logistic regressions capturing the rate of all tasks completed based on exposure to points or no point interfaces (bootstrapped sample).

### S4. Expanding biospecimen collection appointment times

This pilot took place at San Diego Blood Bank (SDBB), a TPC partner. We hypothesize that one reason UBR R/E collection rates increased at scale relative to during the pilot is that there was a smaller proportion of existing UBR R/E donors at SDBB.

### S5. Community partner kit distribution

The following community partners distributed saliva kits at the following locations: Asian Health Coalition (Light and Salt Association of Houston, TX), National Alliance for Hispanic Health (El Centro Hispano of Jonesboro, AR, Nuestra Salud of New Mexico and University of Texas, El Paso), Fifty Forward (Nashville, TN), the Jain Society (Washington D.C.), League of United Latin American Citizens (Washington D.C.), National Hispanic Council on Aging (Washington D.C.), the Denver YMCA, and the Montage Marketing Group.

With in-person pickup, the participant entered the kit barcode into the participant portal to connect the kit with their account (Figure S6). Technical development, design work, and training work were completed. Before the first in-person encounter, community partners attended a TPC-led training session, available in English and Spanish. Informational flyers for event promotion were distributed. During in-person encounters, partners verified participant eligibility and then distributed saliva kits. Participants mailed their kits through the US Postal Service. Standard reminders were also utilized as well as notifications upon the delivery of their kits to the Biobank.


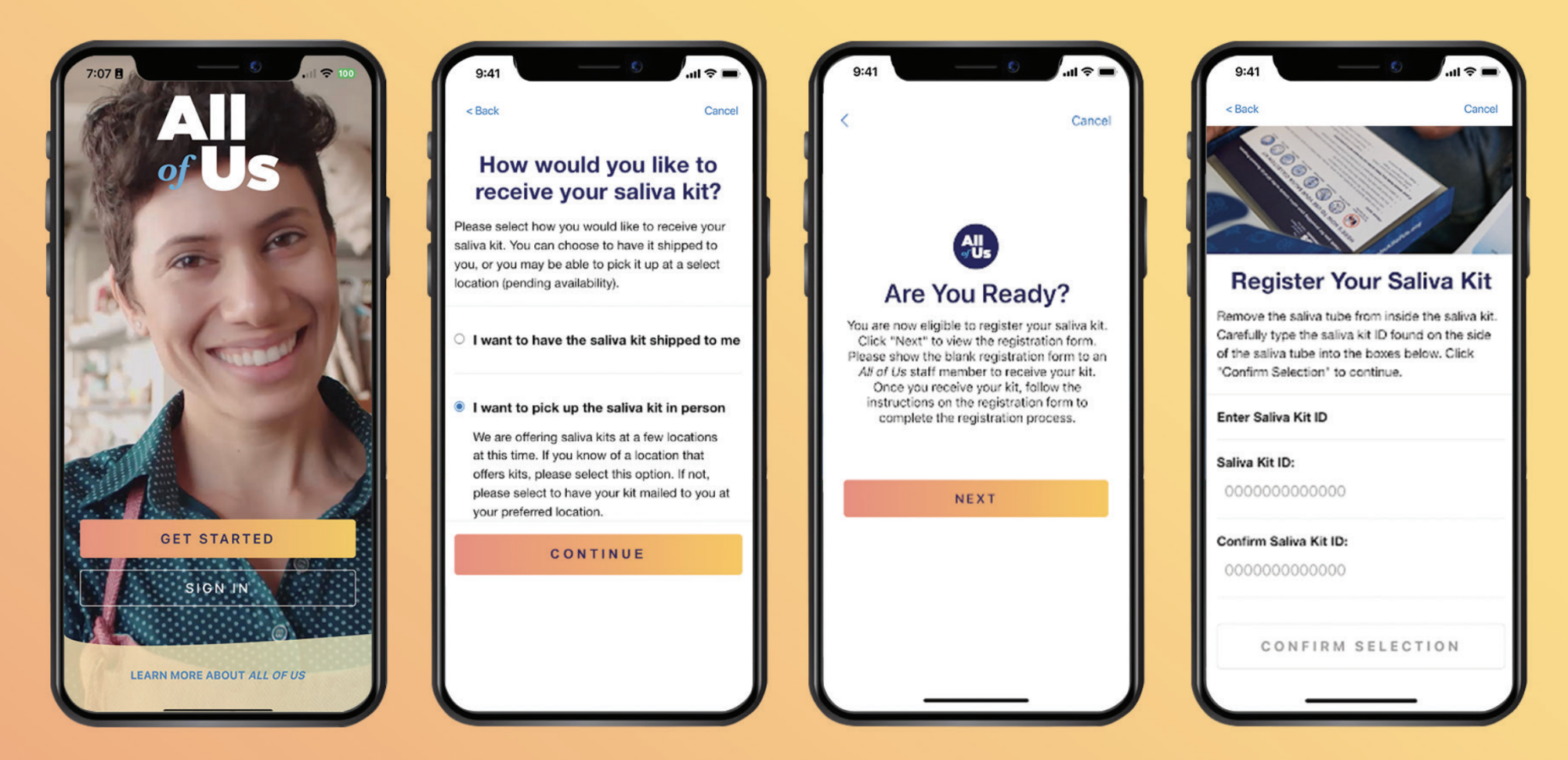


Supplemental Figure 6. Screenshots from the Community Partner Kit Distribution Participant Experience

- - [Link](https://drive.google.com/file/d/17XbXonflmLLUryWAMgF9R0aRWufVHDwP/view?usp=sharing) to event flier
  - [Link](https://drive.google.com/file/d/1-Ujvm9tIq-0OFfyjV9uWp3MoOWYjjY4E/view?usp=sharing) to self-registration one-pager

**References**

1. Evans ND. 9 best practices for better innovation. CIO. October 30, 2020. Accessed July 24, 2024. <https://www.cio.com/article/190908/9-best-practices-for-better-innovation.html>

2. Al-Ubaydli O, List JA, Suskind D. 2017 Klein lecture: The science of using science: Toward an understanding of the threats to scalability. *Int Econ Rev* . 2020;61(4):1387-1409.

3. Al-Ubaydli O, List JA, Suskind DL. What Can We Learn from Experiments? Understanding the Threats to the Scalability of Experimental Results. *Am Econ Rev*. 2017;107(5):282-286.

4. Nosek BA, Ebersole CR, DeHaven AC, Mellor DT. The preregistration revolution. *Proceedings of the National Academy of Sciences*. 2018;115(11):2600-2606.

5. Spieth PM, Kubasch AS, Penzlin AI, Illigens BMW, Barlinn K, Siepmann T. Randomized controlled trials - a matter of design. *Neuropsychiatr Dis Treat*. 2016;12:1341-1349.

6. Simmons JP, Nelson LD, Simonsohn U. False-positive psychology: undisclosed flexibility in data collection and analysis allows presenting anything as significant. *Psychol Sci*. 2011;22(11):1359-1366.

7. Proschan MA, Gordon Lan KK, Wittes JT. *Statistical Monitoring of Clinical Trials: A Unified Approach*. Springer Science & Business Media; 2006.

8. Armitage P, McPherson CK, Rowe BC. Repeated significance tests on accumulating data. *J R Stat Soc Ser A*. 1969;132(2):235.

9. DellaVigna S, Linos E. RCTs to scale: Comprehensive evidence from two Nudge Units. *Econometrica*. 2022;90(1):81-116.

10. Stuart EA. Matching methods for causal inference: A review and a look forward. *Stat Sci*. 2010;25(1):1-21.

11. Powell M, Koenecke A, Byrd JB, et al. A how-to guide for conducting retrospective analyses: example COVID-19 study. *Open Science Framework*. Published online 2020. <https://files.osf.io/v1/resources/3drch/providers/osfstorage/5f5662744f1e5e01426f68a3?format=pdf&action=download&direct&version=2>

12. DellaVigna S, Pope D, Vivalt E. Predict science to improve science. *Science*. 2019;366(6464):428-429.

13. Hallsworth M. A manifesto for applying behavioural science. *Nat Hum Behav*. 2023;7(3):310-322.

14. Vermeulen F, Sivanathan N. Stop doubling down on your failing strategy: how to spot (and escape one before it’s too late). *Harv Bus Rev*. 2017;95(6):110-117.

15. Gallus J, Frey BS. Awards: A strategic management perspective. *Strategic Manage J*. 2016;37(8):1699-1714.

16. Beemer LR, Ajibewa TA, DellaVecchia G, Hasson RE. A Pilot Intervention Using Gamification to Enhance Student Participation in Classroom Activity Breaks. *Int J Environ Res Public Health*. 2019;16(21). doi:[10.3390/ijerph16214082](http://dx.doi.org/10.3390/ijerph16214082)

17. Corepal R, Best P, O’Neill R, et al. Exploring the use of a gamified intervention for encouraging physical activity in adolescents: a qualitative longitudinal study in Northern Ireland. *BMJ Open*. 2018;8(4):e019663.

18. Ahn SJG, Johnsen K, Ball C. Points-Based Reward Systems in Gamification Impact Children’s Physical Activity Strategies and Psychological Needs. *Health Educ Behav*. 2019;46(3):417-425.
